# Effect of interventions to reduce potentially inappropriate use of drugs in nursing homes: a systematic review of randomised controlled trials

**DOI:** 10.1186/1471-2318-11-16

**Published:** 2011-04-17

**Authors:** Louise Forsetlund, Morten C Eike, Elisabeth Gjerberg, Gunn E Vist

**Affiliations:** 1 Norwegian Knowledge Centre for the Health Services, PO Box 7004 St. Olavsplass, 0130 Oslo, Norway; 2 Institute of Medical Genetics, University of Oslo, Oslo, Norway; 3 Section for Medical Ethics, University of Oslo, Oslo, Norway

## Abstract

**Background:**

Studies have shown that residents in nursing homes often are exposed to inappropriate medication. Particular concern has been raised about the consumption of psychoactive drugs, which are commonly prescribed for nursing home residents suffering from dementia. This review is an update of a Norwegian systematic review commissioned by the Norwegian Directorate of Health. The purpose of the review was to identify and summarise the effect of interventions aimed at reducing potentially inappropriate use or prescribing of drugs in nursing homes.

**Methods:**

We searched for systematic reviews and randomised controlled trials in the Cochrane Library, MEDLINE, EMBASE, ISI Web of Knowledge, DARE and HTA, with the last update in April 2010. Two of the authors independently screened titles and abstracts for inclusion or exclusion. Data on interventions, participants, comparison intervention, and outcomes were extracted from the included studies. Risk of bias and quality of evidence were assessed using the Cochrane Risk of Bias Table and GRADE, respectively. Outcomes assessed were use of or prescribing of drugs (primary) and the health-related outcomes falls, physical limitation, hospitalisation and mortality (secondary).

**Results:**

Due to heterogeneity in interventions and outcomes, we employed a narrative approach. Twenty randomised controlled trials were included from 1631 evaluated references. Ten studies tested different kinds of educational interventions while seven studies tested medication reviews by pharmacists. Only one study was found for each of the interventions geriatric care teams, early psychiatric intervening or activities for the residents combined with education of health care personnel. Several reviews were identified, but these either concerned elderly in general or did not satisfy all the requirements for systematic reviews.

**Conclusions:**

Interventions using educational outreach, on-site education given alone or as part of an intervention package and pharmacist medication review may under certain circumstances reduce inappropriate drug use, but the evidence is of low quality. Due to poor quality of the evidence, no conclusions may be drawn about the effect of the other three interventions on drug use, or of either intervention on health-related outcomes.

## Background

Several studies have shown that the prescription rate and consumption of drugs in nursing homes are high and that prescribing often is inappropriate [1-6]. Inappropriate use or prescribing of drugs comprises over-use as well as underuse of drugs, prescribing of multiple drugs with known interactions, for the wrong indication or in wrong doses or for too long duration. Appropriateness of prescribing can be assessed by validated tools for reviewing drug utilisation [7]. As much as 40% of prescriptions for residents in nursing homes may be inappropriate [6]. Of particular concern is the high consumption of psychotropic drugs, sedatives and sleep medication. Residents in nursing homes often have a complex and complicated illness profile ranging from simultaneous occurrence of several chronic diseases, depression, pain and sleep problems. The majority of residents in nursing homes also suffer from dementia, with the psychiatric and behavioural symptoms this often entails [3]. For these reasons, many residents use several drugs simultaneously, with increased risk of interactions between drugs, adverse effects and medication errors, with the possibility of increased morbidity and mortality. It is, therefore, desirable to reduce potentially inappropriate drug use. This review is an update of a Norwegian systematic review in Norwegian commissioned by the Norwegian Directorate of Health [8]. The objective for this systematic review was to identify, assess and summarise available scientific evidence about the effect of interventions that could be used to reduce potentially inappropriate use of drugs in nursing homes.

## Methods

### Search strategy

A search strategy was developed on the basis of the inclusion criteria and included the following databases: The Cochrane Library, MEDLINE, EMBASE, ISI Web of Knowledge and the Database of Abstracts of Reviews of Effects (DARE) and Health Technology Assessment Database (HTA) at the Centre for Reviews and Dissemination (CRD). MEDLINE and EMBASE was searched from their inception to August/September 2009 for systematic reviews and for randomized controlled trials for the last five years to compensate for the time lag in the Cochrane Central Register of Controlled Trials. No language restrictions were added to the search algorithms. We only searched for published literature. All searches were updated in April 2010. The complete search strategy is presented in Additional file 1, Table S1.

### Inclusion criteria

Inclusion criteria were all studies of interventions aimed wholly or partly at reducing potentially inappropriate use or prescribing of drugs for elderly people in nursing home settings and that measured drug use. Studies were only included if the primary outcome, drug use, was assessed by explicit or implicit criteria [7] or where specific drugs were targeted for reduction as defined by authors. Secondary outcome measures were falls, hospital admission, physical restraints and mortality. Comparison interventions were care as usual or other interventions. Study design inclusion criteria were high quality systematic reviews of randomised controlled trials and/or primary studies with a randomised controlled design. For a review to be considered systematic, three criteria had to be fulfilled: a documented systematic search strategy, critical appraisal of included studies by at least two independent reviewers and taking the quality of evidence into consideration when drawing conclusions. Inclusion criteria for language were Norwegian, Swedish, Danish, Finish, English or German.

### Exclusion criteria

Studies of the effect of withdrawal of drugs and studies that otherwise did not satisfy all the inclusion criteria were excluded.

### Selection of studies

Two reviewers (LF, EG; LF, MCE) screened references and abstracts independently for identification of studies that potentially satisfied the inclusion criteria. If one reviewer assessed a reference to be potentially relevant, the full-text article was ordered. Each full-text report was assessed independently by two reviewers (LF, MCE) for inclusion or exclusion.

### Assessment of risk of bias

Two reviewers (LF, MCE) assessed risk of bias for results of included randomized controlled studies using the Cochrane Collaboration's "Risk of Bias Table" [9]. In cases of disagreement, a third reviewer (GEV) was involved until consensus was reached. Each outcome within a study was assessed as of low, unclear or high risk of bias, using summary evaluation according to the table. When there was unclear risk of bias for key domains, the summary risk of bias for the outcome was also assessed as unclear. For cluster trials we added three more domains to be assessed: baseline balance, blinding of recruiters, and completeness of data for clusters and health personnel. In addition, under biases defined as 'other' in the table, we assessed the unit of analysis. If matching had been used, we assessed whether this had been adjusted for in the analysis. When clusters are allocated simultaneously, concealment is not regarded as an issue. Accordingly, if it was unclear whether clusters had been allocated all at once, we rated concealment of allocation as 'unclear'.

### Data extraction, data synthesis and grading of evidence

When possible, we collected or calculated the relative risk (RR) for dichotomous outcomes and mean difference between the groups with confidence intervals for continuous outcomes. No meta-analysis was conducted, partly because the execution, intensity and duration of these types of interventions are very different and partly because the outcome was measured in several different ways and with different follow-up periods. We therefore made a qualitative analysis and presented the data in tables. The following data were extracted: participants, intervention description and the degree of implementation, comparative interventions and outcomes.

The quality of the complete documentation of each of the primary outcomes was assessed by GRADE (Grading of Recommendations, Assessment, Development and Evaluation: http://www.gradeworkinggroup.org).

## Results

**Figure 1:**
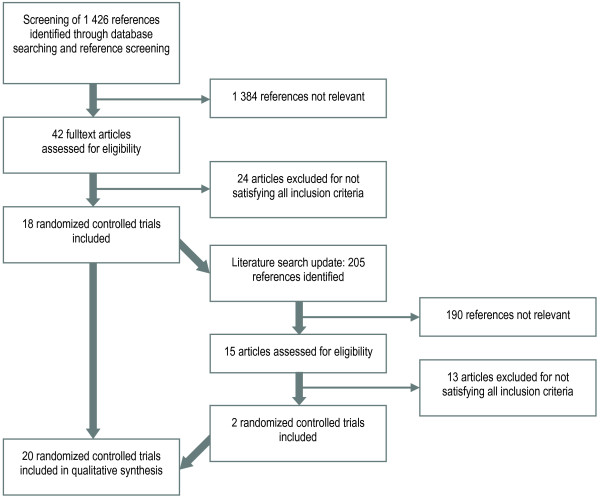
**Flow chart of study selection**.

We identified and assessed 1631 references (205 of these from the update search) by title and abstract according to inclusion and exclusion criteria. Of these, 57 references (15 from the update) were further evaluated for inclusion in full-text. Twenty-one publications (2 from the update) of 20 randomised controlled trials met the inclusion criteria [10-30], 15 of which were randomized by clusters. A flow diagram of the selection process is presented in Figure 1. None of the 19 identified review articles were included in this paper, as they either failed to meet the inclusion criteria of high quality systematic reviews or because they reviewed studies of elderly in general. Detailed reasons for exclusions of reviews and randomised controlled trials are reported in 'Excluded Studies' in Additional file 1, Table S2.

### Risk of bias in included studies

The results in one study were assessed to have a low risk of systematic bias, in five studies to have a high risk and in the remaining 14 studies to have an unclear risk of systematic bias (Additional file 1, Table S3). The most common reasons for risk of bias were unclear information on the generation of the randomization sequence and/or concealment of allocation and incomplete follow-up of residents. In none of the cluster randomized studies did the authors explicitly report whether the persons recruiting residents for the trial were blinded to group allocation. Two of these studies were also assessed as having unit of analysis errors in their analyses (McCallion 1999 [21], Schmidt 1998/Claesson 1998 [26]).

### Outcomes

In three studies the quality of drug prescribing was assessed by using the Medication Appropriateness Index (MAI) (Crotty 2004b [14], Crotty 2004c [15]) or a similar scoring tool developed for use in the study (Avorn 1992 [10]). MAI is a validated measure that assesses ten criteria for each medication: Indication, effectiveness, dosage, correct directions, practical directions, drug-drug interactions, drug-disease interactions, duplication, duration and cost [7]. A weighted score is generated to express the degree of prescribing appropriateness. Four other studies evaluated the appropriateness of drugs according to explicit criteria, using guidelines from the Swedish Medical Product Agency (Schmidt 1998 [26]/Claesson 1998 [12]), guidelines issued in connection with OBRA (US Ombudsman Reconciliation Act) (Furniss 2000 [17]), Beers' criteria (Midlöv 2002 [23]) or a novel algorithm developed for the study purpose (Patterson 2010 [29]). Beers' criteria are a list of drugs that should be avoided in the elderly in general or in elderly with specific disorders and gives indications of maximum doses [31]. Reduction in the overall number of drugs prescribed were measured in two studies (Cavallieri 1993 [11], Zermansky 2006 [28]) while the remaining 11 studies measured reduction in prescription of targeted classes of drugs. The follow-up period in all studies ranged from 1 month to 12 months (details are given in GRADE tables).

### Categorisation of interventions

We classified the interventions into seven categories on the basis of their main component:

• Educational outreach initiatives (2 studies)

• Educational meetings (5 studies)

• Educational meetings with at least one additional intervention (3 studies)

• Medication review (7 studies)

• Geriatric assessment and care teams (1 study)

• Early psychiatric intervention (1 study)

• Activity program interventions for residents (1 study)

All interventions were compared to usual practice.

### Educational outreach interventions

Two studies conducted in 32 nursing homes with a total of 1538 residents examined the effect of educational outreach interventions (Avorn 1992 [10]; Crotty 2004a [13]). A description of the participants, interventions and outcomes is presented in Table 1. An external clinical pharmacist visited the physicians that cared for the residents in the intervention nursing homes to provide information on appropriate drug use. The pharmacist in Crotty 2004a [13] provided audit information on falls and psychotropic medication in the visit to the physician and also made a visit to the nursing homes to speak to the staff. The intervention in Avorn 1992 [10] included four educational meetings with nurses and assistant nurses in addition to the outreach visit, and may therefore have been more intensive.

**Table 1:** Description of included primary studies for the comparison educational outreach versus usual practice

Study	Participants	Intervention	Comparison	Outcomes
**Avorn 1992 **[10]	Physicians, nurses and nursing assistants in 6 nursing homes with 431 residents in the experiment group and 6 nursing homes with 392 residents in the control group in the US. Age: Not reported.	Aimed to reduce the excessive use of sedating drugs. Three interactive educational outreach visits by a pharmacist to nursing home physicians to reduce the use of psychoactive drugs. Only physicians that exceeded a treshold value for psychoactive drug prescribing at the baseline evaluation were targeted. Six literature summaries done by research team on e.g. management of insomnia, confusion and agitation were disseminated to all physicians in three mailings and used as discussion aids in the educational visit. Four training sessions were held for nurses and nursing assistants in separate groups on patient care, alternatives to psychoactive drugs and adverse effects. **Extent of implementation**: Not reported.	Usual care.	Score for use of psychoactive drugs, proportion of residents using antipsychotics.

**Crotty** **2004a **[13]	61 physicians, nurses and nursing assistants in 10 nursing homes with 381 residents in the experiment group and 37 physicians with other health personnel in 10 nursing homes with 334 residents in the control group. Mean age: 84 years.	Aimed at implementing evidence based practice in residential care. Doctors received two 30 minute educational outreach visits by a pharmacist in their offices. The risks of psychotropic drug use was one of several other key messages, combined with detailed audit information on fall rates, psychotropic prescribing patterns and stroke risk reduction practices in the nursing home of each physician. A link nurse was appointed at each facility. The link nurses were trained in four two hour sessions in which medication management was one of the topics. Also, a pharmacist visited each nursing home and spoke to staff about reducing the use of psychotropic medication. **Extent of implementation**: Not reported.	Usual care	Percentage of residents prescribed and administered any psychotropic medication, percentage of residents who had a fall incident in a 3 month period.

### Drug use

A score for psychoactive drug use was developed in Avorn 1992 [10] to assess the appropriateness of drug use. At post-test the mean difference in favour of the intervention group was -0.37 scores (95% CI -0.08 to -0.67) and 18% fewer residents compared to the control group used antipsychotic drugs (95% CI -3 to -33%).

Crotty et al. 2004a [13] measured the prescription rate for psychotropics. The relative risk for having psychotropics prescribed was not statistically significant (RR 0.89 95% CI 0.69 to 1.15), neither was the number of regularly used psychotropics (RR 0.93 95% CI 0.82 to 1.05).

### Health-related outcomes

No statistically significant difference in number of falls between the two groups during the previous three months was reported (Crotty 2004a [13]): RR 1.17 (95% CI 0.86 to 1.58). None of the other health-related outcomes were measured.

### Summary and quality of evidence

Educational outreach combined with education of key health personnel may in some circumstances lead to a small reduction in inappropriate drug use. However, the quality of evidence for this result varies from low to very low (GRADE summary of findings in Additional file 1, Table S4). Number of falls was the only health-related outcome measured, but the quality of the evidence was too low to draw any conclusions.

### Educational meetings interventions

Five studies conducted in 32 nursing homes with a total of 811 residents examined the effect of educational meetings in the work setting (Fossey 2006 [16], Kuske 2009 [19], McCallion 1999 [21], Stein 2001 [27], Testad 2010 [30]) (details in Table 2). The educational interventions were heterogeneous both in contents, e.g. communication skills training and training in person-centred care, in intensity of the intervention and in measurements. However, all educational activities seem to have been of low intensity, with maximum duration of 13 hours (Kuske 2009 [19]) or two days (Testad 2010 [30]). One study only stated having carried out a 10-month training intervention without specifying the number of hours (Fossey 2006 [16]).

**Table 2:** Description of included primary studies for the comparison educational meetings and workshops versus usual care

Study	Participants	Interventions	Comparison	Outcomes
**Fossey** **2006 **[16]	Health personnel in 6 nursing homes with 181 residents in the intervention group and 6 nursing homes with 168 residents in the control group Median age: 82 years (range 53-101).	Aimed at reducing the prescribing of neuroleptics for residents with dementia. Ten month educational program delivered by a psychologist, occupational therapist or nurse with a focus on alternatives to drugs for managing agitated behaviour in dementia. The staff received training in providing person centered care and to develop skills through didactic instruction, skills training and weekly supervision and follow up both in groups and individually. The programme entailed a 'systemic consultation approach'. **Extent of implementation**: Not reported.	Usual care.	Percentage of residents using neuroleptics, percentage of residents with at least one fall.

**Kuske 2009 **[19]	Two nursing homes with 89 caregivers and 68 residents allocated to the intervention group and two nursing homes with 94 caregivers and 74 residents allocated to the control group, in Germany. Mean age: 81 years.	Aimed at increasing the caregivers' knowlegde and competencies for dealing adequately with residents with dementia and reducing the number of residents who were given sedative drugs and/or being physically restrained. A training program that consisted of five modules (13 one hour educational meetings in a 13 week intervention period) was developed using focus groups and review of international literature to find the key problem areas. The aim was to improve the interaction between caregivers and residents by improving caregivers knowledge and expertise in providing care to residents with dementia. Lectures were given by health care researchers with practical experience in nursing in small groups of max 12 participants. Didactic teaching methods were used for the theoretical introduction and problem based learning methods for practical learning and skills training. **Extent of implementation**: Caregivers that did not attend at least 10 sessions (i.e. 40% of caregivers) were excluded.	Control 1: A group that was on the waiting list. Control 2: A group of health professionals who received relaxation. This comparison has not been included here.	Percentage of residents using sedative drugs, percentage of residents exposed to physical restraint.

**McCallion 1999 **[21]	39 nursing assistants in one unit in one nursing home with 49 residents in the intervention group and 49 nursing assistants in one unit in one nursing home with 56 residents in the control group in USA. Mean age: 84 years.	Aimed at improving the well being of nursing home residents with dementia by means of a communication skills program for nursing assistants. Education delivered by a master's level social worker with practical experience. Alternately group instruction in small groups five times à 45 minutes and individual conferences à 30 minutes four times (for skills training and feedback). The program addressed four areas: knowledge of dementia, verbal and nonverbal communication, memory aids and problem behaviours. **Extent of implementation**: Not reported.	Usual care for the control group in the 6 months that the intervention lasted for the experimental group. Then, the control group also received the same intervention for 3 months and both groups were measured again at 9 months (we used data from the 6 month measurements).	Number of days the previous week that residents used psychotropic drugs, number of days the previous week that restraints were used.

**Stein 2001 **[27]	Health personnel and 76 residents aged 65 years and more taking non steroidal anti-inflammatory drugs (NSAIDs) regularly in 10 nursing homes in the intervention group and 71 residents taking NSAIDs regularly in 10 nursing homes in the control group in USA. Age: ≥65 years.	Aimed at reducing the use of NSAIDs in the management of osteoarthritis in nursing home residents. The educational programme focused on alternative approaches to NSAIDs to relieve muscle and joint pains in residents, e.g. use of acetaminophen. One meeting with both the administrative and professional management and a separate meeting with each nursing home study coordinator to review the purpose and educational materials during the initial phase of the project, a 30 minute structured teaching session for physicians and nursing staff. **Extent of implementation**: On average, in each nursing home it was estimated that 60-65% of all staff received the educational programme.	Usual care	Use of NSAIDs and acetaminophen the past seven days.

**Testad 2010 **[30]	197 care staff. Two nursing homes with 113 residents in the intervention group and two nursing homes with 98 residents in control. Median age 86 years.	Aimed at reducing agitation in residents with dementia, use of restraints and antipsychotic drugs. Relation Related Care education and training program, consisting of predisposing, enabling and reinforcing elements. Organized as a two day seminar and monthly group guidance for six months. **Extent of implementation**: "All care staff with and without formal education, including leaders and domestic staff, participated... [...] During the study period, there was considerable turnover of staff...": 56 (54%) remained in intervention and 53 (57%) in the control group.	Usual practice.	Percentage of residents taking antipsychotic drugs, use of restraint.

### Drug use

After ten months training and support for the health personnel in person centred care and the use of alternatives to drugs for agitated behaviour in dementia, the weighted mean difference in favour of the intervention group for reduced use of neuroleptics was 19.1% (95% CI 0.5% to 37.7%) (Fossey 2006 [16]). There was no statistically significant difference between groups for use of other psychotropic drugs (weighted mean difference -5.9% (CI -27.2% to 5.5%)).

Residents' use of sedative drugs after a 13-week small groups training program targeted at all nursing home staff members was examined in another study (Kuske 2009 [19]). The program was based on focus groups and a literature study. The use of sedative drugs in the intervention group was not statistically significantly different from the use in the control group at six months.

Psychotropic drug use was measured in a study of a communication skills program for nursing assistants (McCallion 1999 [21]). At six months the residents in the intervention group received psychotropic medication for an average of 1.30 days (SD 2.15) compared to the control group average of 1.57 (SD 1.71) days during the previous week. The difference was not statistically significant.

Use of non-steroidal anti-inflammatory drugs (NSAIDs) and use of acetaminophen as the preferred drug was measured at three months in an educational program for physicians and other staff (Stein 2001 [27]). The mean difference between groups for the use of NSAIDs was -4.3 days less use (95% CI -6.41 to -2.19) in the intervention group for the last seven days. The mean difference between groups for the preferred drug acetaminophen, in favour of the intervention group, was 3.0 days (95% CI 1.53 to 4.47) for the last seven days.

The proportion of residents using antipsychotic drugs was measured in a study of the educational and training program Relation-Related Care (Testad 2010 [30]). In the intervention group 29% used antipsychotics immediately after the intervention and 32% six months later versus 14% and 9% respectively in the control group. These differences were not statistically significant (no confidence intervals reported).

### Health-related outcomes

The proportion of residents with at least one fall in the past 12 months was measured in one of the studies (Fossey 2006 [16]). There was no statistically significant difference between the two groups (weighted average difference 2.6% (95% CI -18.7 to 23.8)). Use of physical restraint (bed fences, chair that prevents ascension, etc.) was measured in three of the studies. In the study by Kuske 2009 [19] results were reported as coefficients with standard deviations. The use of restraints increased significantly more in the control group compared to the intervention group (p = 0.045 when comparing change in the groups as the number of residents who had been subject to physical control during the study sequence). In the study by McCallion 1999 [21] no statistically significant differences between the groups were recorded: 1.88 (SD 1.82) days of physical restraint in the preceding week in the intervention group compared to 1.75 (SD 1.42) in the control group. The third study reported no statistically significant difference between groups for 'interactional' physical restraint (force or pressure in medical examination, treatment or in activities of daily living) (Testad 2010 [30]). Immediately after the intervention period 48% of residents in the intervention group had been subjected to physical restraint versus 46% in the control group.

### Summary and quality of evidence

Educational meetings may in some circumstances lead to a small reduction in use of drugs in nursing homes. However, the evidence for this result varies from low to very low quality (GRADE summary of findings in Additional file 1, Table S5). The quality of the evidence for the effect of these interventions on health-related outcomes is too low to draw any conclusions.

### Educational meetings with at least one additional intervention

We included three studies conducted in 88 nursing homes with 8599 residents that used at least one more intervention in addition to educational meetings (Loeb 2005 [20], Meador 1997 [22], Roberts 2001 [24]) (details in Table 3). The main additional intervention in all studies was educational outreach. One of the studies also had a partial medical review with recommendations to physicians (Roberts 2001 [24]).

**Table 3:** Description of included primary studies for the comparison educational meetings and workshops with other co-interventions versus usual care

Study	Participants	Interventions	Comparison	Outcomes
**Loeb 2005 **[20]	Nurses and physicians, 2156 residents in 12 nursing homes participated in the intervention group and 2061 residents in 12 nursing homes partcipated in the control group in USA and Canada. Age not reported.	Aimed at reducing the number of prescriptions for antimicrobials for suspected urinary tract infections. Multifaceted intervention: Diagnostic and treatment algorithm for urinary tract infections introduced to physicians and nurses, small group interactive sessions with case scenarios for nurses, videotapes, outreach visits to the physicians that cared for 80% or more of the residents, visits from the researchers every three months to address any questions, one nurse in each nursing home appointed to remind nurses to use the algorithm. **Extent of implementation: **Not reported.	Usual care.	Number of amtimicrobial prescriptions for suspected urinary tract infection per 1000 resident days, total number of prescriptions for amtimicrobials per 1000 resident days, number of admissions to hospital, mortality.

**Meador** **1997 **[22]	Health personnel and 575 residents in 6 nursing homes in the intervention group and 577 residents in 6 nursing homes in the control group in USA. Age: ≥65 years	Aimed at reducing antipsychotic use in nursing homes with high use rates. Physicians, nurses, nursing assistants and other direct care staff were trained to use structured guidelines. Educational outreach: A geropsychiatrist visited all physicians who had five or more patients in the home to dicuss risks and benefits of antpsychotics and delivered printed material. Educational meetings: A trained nurse educator conducted five to six 1 hour inservice programs (including case examples, role playing and problem solving sessions) for staff over a 1 week period. Four weeks after the inservice programs were completed, a follow up session was conducted for the staff. Further consultations and meetings could be arranged if requested (it is not reported if it was). **Extent of implementation: **Not reported.	Usual care and waiting list.	Use of antipsychotics as registered in the medication administration records.

**Roberts 2001 **[24]	Nurses, 905 residents in 13 nursing homes in the intervention group and 2325 residents in 39 nursing homes in Australia. 70% of residents ≥80 years	Aimed at changing drug use, mortality and morbidity. 12 months intervention involving three phases: introducing a new professional role to stakeholders with relationship building, nurse education and medication review by pharmacists. In focus groups, written and telephone communication and face to face professional contact between nursing home staff and clinical pharmacist drug policies and resident problems were dicussed. 6-9 problem based education sessions (11 hours total) were held for nurses. The subjects were geriatric pharmacology, depression and dementia, incontinence, falls, insomnia, constipation, and pain supported by wall charts, bulletins, telephone calls and visits by clinical pharmacists (average contacts per nursing home was 26 h). For 500 selected residents clinical pharmacists wrote down the results of their review of medication, which was then discussed with the nurses and included in each patient's record and thus made available to the residents' physician. **Extent of implementation: **Not reported.	Usual care.	Percentage of residents being administered psychotropic medication, mortality, number of hospitalisations.

### Drug use

The number of prescriptions for antibiotics for suspected urinary tract infection per 1000 resident days was reported in a study of interactive teaching of nurses, combined with procedural tools and educational outreach (Loeb 2005 [20]). Although the weighted mean difference was statistically significant at 12 months (-0.49 prescriptions per 1000 resident days (95% CI -0.93 to -0.06)), the difference in total number of prescriptions for antibiotics was not (-0.37 prescriptions per 1000 resident days (95% CI -1.17 to 0.44)).

Use of antipsychotics at 6 months was measured in a study of an educational program combined with educational outreach visits (Meador 1997 [22]). The mean difference in consumption of antipsychotics was statistically significant in favour of the intervention group for the last preceding 30-day period (-6.30 days per 100 resident days (95% CI -6.55 to -6.05)).

The proportion of residents that used psychotropic drugs was measured in a study that examined the effect of a year long clinical pharmacy program involving development of professional relationships, nurse education on medication issues, and individualised medication reviews (Roberts 2001 [24]). The result was not statistically significant (RR 0.91 (95% CI 0.83 to 1.00) at 12 months.

### Health-related outcomes

Two studies measured mortality and number of admissions to hospital (Loeb 2005 [20], Roberts 2001 [24]), but no statistical significant differences between groups were found.

### Summary and quality of evidence

Educational meetings with at least one additional intervention may in some circumstances lead to a small reduction in use of drugs in nursing homes. However, the evidence for this result is of low quality (GRADE summary of findings in Additional file 1, Table S6). No statistically significant effects were demonstrated for number of hospitalisations or mortality and the quality of the evidence for these results is too low to draw any conclusions.

### Medication review

We included seven studies conducted in 255 nursing homes with 3212 residents that examined the effect of medication review with a pharmacist (Furniss 2000 [17], Zermansky 2006 [28], Schmidt 1998 [26], Crotty 2004b [14], Crotty 2004c [15], Patterson 2010 [29], Midlöv 2002 [23]) (details in Table 4). Reviews were made either by the pharmacist alone (Furniss 2000 [17]; Zermansky 2006 [28]), as part of a team (Schmidt 1998 [26]; Crotty 2004b [14]; Crotty 2004c [15]), with the pharmacist discussing the results of the medical review with nursing staff and physicians separately (Patterson 2010 [29]) or together with other specialists, resulting in a letter to the residents' physician (Midlöv 2002 [23]). In the latter study, it was unclear whether these specialists were external experts or were working at the nursing home.

**Table 4:** Description of included primary studies for the comparison medical review by pharmacist versus usual care

Study	Participants	Intervention	Comparison	Outcomes
**Crotty** **2004b **[14]	Physicians, registered nurses, 56 residents in the intervention group and 54 residents in the control group that were assigned to 85 long term care facilities in Australia. Mean age: 83 years.	Aimed at improving medication management services by transferring information on medications to care providers in the longterm care facilities. The transition pharmacist compiled a medication transfer summary and faxed this to the family physician and the community pharmacist. The pharmacist coordinated an evidence based medication review that was to be performed by the community pharmacist contracted to the facility within 14 days of the transfer. After this, the transition pharmacist also arranged and participated in a case conference with the family physician, the community pharmacist and a registered nurse at the facility within a month of the transfer. **Extent of implementation**: Medical review was performed for 36 residents (64%). Case conferences took place for 8 residents (14%).	Usual care: Standard hospital discharge summary.	Medication Appropriateness Index score, falls, hospital admissions (emergency visits and readmissions).

**Crotty** **2004c **[15]	General practitioners, geriatricians, pharmacists, residential care staff, 50 residents in 5 nursing homes in the intervention group and 50 residents in 5 nursing homes in control group 1 and 54 residents in control group 2 (not included here) in Australia. Mean age: 85 years.	Aimed at improving appropriateness of medications. Two multidisciplinary case conferences (GP, geriatrician, pharmacist, residential care staff, representative of the Alzheimer's Association of South Australia) were conducted 6-12 weeks apart. A medical review was prepared beforehand by the resident's GP. **Extent of implementation: **Not reported.	Usual care. Both groups received a half day education in how to handle behaviour problems in residents with dementia.	MAI (Medication Appropriateness Index) score, number of drugs.

**Furniss** **2000 **[17]	158 residents in 7 nursing homes in the intervention group and 172 residents in 7 nursing homes in the control group in United Kingdom. Mean age: 81 years.	Aimed at reducing the number of prescribed drugs. The pharmacist collected details of current medication for each resident from the Medicines Administration Record chart in each home, compiled a brief medical history and made the staff identify any current problems. The pharmacist checked whether the use of neuroleptics were in accordance with the US Ombudsman Reconciliation Act guidelines and made suggestions for change of medication if necessary. Three weeks afterwards, the homes were revisited to identify any immediate problems and to check on whether changes had been implemented. **Extent of implementation**: Not reported.	Usual care.	Mean number of prescribed drugs.

**Midlöv 2002 **[23]	41 residents with epilepsy in the intervention group and 33 residents in the control group; 51 residents with Parkinson in the intervention group and 33 residents in the control group in 48 nursing homes in Sweden. Mean age: 80 years.	Aimed at improving the pharmacological treatment. Pharmacists reviewed and documented the patients' drug use and any problems related to the drug use as reported by the residents, their contact person at the nursing home and the resident's physician. A multidisciplinary team consisting of the data collecting pharmacist, a pharmacist with a special experience in neurology, a primary care physician, neurologist, neuropsychiatrist and a clinical pharmacologist discussed the collected information and made suggestions. A list of recommended changes in medication was sent to the resident's physician. **Extent of implementation**: Not reported.	Usual care.	Number of drugs.

**Patterson 2010 **[29]	11 nursing homes with 173 residents in intervention group and 11 nursing homes with 161 residents in control group. Mean age: 83 years.	Aimed at reducing inappropriate prescription of psychoactive medications and falls. Specially trained study pharmacists visited nursing homes monthly for 12 months. Information for each resident was collected from the nursing home record, the GP and from the local community pharmacist if needed. The residents themselves, their nurses and next of kin were interviewed to assess residents' need for medication. An algorithm were used by pharmacists for guidance in assessing the inappropriateness of psychoactive medication. Recommendations were discussed with nursing staff. Meetings were held with the residents' GP to discuss and decide about medication and feedback were given to nursing staff. **Extent of implementation**: Not reported.	Usual care.	Proportion of residents prescribed one or more inappropriate psychoactive (anxiolytic, hypnotic or antipsychotic) drugs, change in number of inappropriate psychoactive drugs, rate of falls per 100 resident months.

**Schmidt** **1998 **[26]/**Claesson** **1998 **[12]	626 residents in 15 nursing homes in the intervention group and 1128 residents in 18 nursing homes in the control group in Sweden. Mean age: 83 years.	Aimed at minimising the use of nonrecommended drugs as defined by guidelines from the Swedish Medical Product Agency. One pharmacist was appointed from the local pharmacy to spend one day per month in a nursing home. The pharmacist attended two training sessions in geriatrics, drug use and in interdisciplinary collaboration methods before the intervention and three sessions during the 12 months intervention. The appointed pharmacists helped in organising monthly multidisciplinary meetings to discuss and improve the use of drugs that could cause confusion and memory impairment. A physician, pharmacist and selected nurses and nursing assistants participated in discussing each resident's drug use. The length of the meetings was locally adapted. Pharmacists formed regional networks to support their function in the project. **Extent of implementation**: Not reported.	Usual care.	Number of prescriptions for any psychotropic, antipsychotics, hypnotics, anxiolytics, antidepressants.

**Zermansky 2006 **[28]	661 residents on one or more drugs selected from 65 nursing homes in United Kingdom: 331 allocated to the intervention group and 330 to the control group. Mean age: 85 years.	Medical review by a pharmacist by using the patient's clinical record and by consultation with the patient and carer. On this basis, the pharmacist made recommendations and forwarded them on a written proforma to the GP for acceptance and implementation. The GPs indicated acceptance by ticking a box on the proforma. **Extent of implementation**: Not reported.	Usual care.	Number of changes in medication per resident, total number of drugs used.

### Drug use

Number of drugs prescribed was used to measure the effect of a medication review in one study (Furniss 2000 [17]). The mean difference between groups at eight months, adjusted for baseline differences, was not statistically significant (0.5 prescriptions (95% CI -0.04 to 1.0; p = 0.07).

In Zermansky 2006 [28] the effect of the medication review was measured as mean number of drug changes. Although a statistically significant difference in favour of the intervention group was found at six months (ratio of means 1.34 (95% CI 1.21 to 1.48)), there were no statistically significant differences between groups for the total number of drugs used (ratio of means 0.98 (95% CI 0.92 to 1.04).

The total number of prescriptions for any psychotropic drug was measured in a study investigating the effect of regular multidisciplinary team meetings (Schmidt 1998 [26]). Each resident's medication was discussed in pursuance of the guidelines from the Swedish Medical Product Agency. A pharmacist organised and participated in the meetings. The difference in total number of prescriptions for any psychotropic drug was not statistically significantly different between the groups at one month follow-up (RR 0.97 (95% CI 0.92 to 1.03)). The authors also analysed use of the various kinds of psychotropic drugs: antipsychotics, hypnotics, anxiolytics and antidepressants. However, change was reported for each group separately and not compared between the groups, which make it difficult to know the effect estimate for use of each drug group. Because of baseline differences, dependency between baseline and post intervention values and the cluster design, it was not appropriate to reanalyse on the basis of the summary statistics presented in the paper.

Two studies by Crotty and colleagues measured Medication Appropriateness Index (MAI) scores after medication review by a pharmacist working in a team. In Crotty 2004b [14], the mean difference in MAI score at eight months follow-up was -4 (95% CI -6.76 to -1.24) in favour of the intervention group. However, the difference was due to a worsening in the MAI score in the control group whereas the score in the intervention group did not change. In Crotty 2004c [15], The MAI score improved significantly in the intervention group compared to the control group (4.1 (95% CI 2.1 to 6.1) versus 0.4 (95% CI -0.4 to 1.2); p < 0.001) at three months after start of the intervention. However, there was no statistical significant difference between groups in number of drugs used.

In Patterson 2010 [29], an algorithm was used to decide the inappropriateness of psychoactive medication. The difference in proportion of residents taking psychoactive drugs at 12 months was statistically significant, 20% in the intervention group versus 50% of residents in the control group (OR 0.26 (95% CI 0.14 to 0.49)).

Use of drugs by residents with Parkinson's disease or residents with epilepsy was measured in a study that examined the effect of a combined pharmacist/specialist medication review (Midlöv 2002 [23]). No statistically significant differences in change scores were found between intervention and control groups. The change score in the intervention group was 0.19 drugs versus 0.56 for the control group (p = 0.26) in residents with epilepsy and -0.14 drugs versus 0.07 drugs (p = 0.88) in residents with Parkinson's disease.

### Health-related outcomes

Number of falls during the intervention period was measured in three of the studies (Crotty 2004b [14], Furniss 2000 [17], Patterson 2010 [29]) and number of patients that fell was measured in a fourth study (Zermansky 2006 [28]). None of them found any statistically significant effects. There was, however, a statistically significant reduction of falls per resident in the intervention group in one study measuring this (ratio of means of 0.59 (95% CI 0.49 to 0.70)) (Zermansky 2006 [28]).

Number of hospital admissions was measured in two of the studies (Zermansky 2006 [28], Crotty 2004b [14]). When data for deceased residents were included, there were no statistically significant differences between groups at eight weeks in the study by Crotty 2004b [14] (RR 0.58 (95% KI 0.28 to 1.21)). Neither was any statistically significant results for being admitted to hospital during a 6-month period reported in Zermansky 2006 [28] (OR 0.89 (95% CI 0.56 to 1.41)).

Two of the studies also measured mortality (Furniss 2000 [17], Zermansky 2006 [28]). In Furniss 2000 [17], mortality was lower in the intervention group for the intervention period, but not for the study period: 4 deaths in the intervention group versus 14 deaths in the control group (p = 0.028). No statistically significant difference in mortality during the last six months was reported in Zermansky 2006 [28] (OR 0.89 (95% KI 0.56 to 1.41)).

### Summary and quality of evidence

Medication review by a pharmacist in teamwork with other health personnel may in some circumstances lead to a small change in use of drugs in nursing homes. However, the quality of the evidence for this result varied from low to very low (GRADE summary of findings in Additional file 1, Table S7). Only one of the studies indicated both a statistically significant change in use of drugs and a positive change in number of falls per resident. However, the quality of the evidence for all of the measured health-related outcomes; use of hospital services, falls or mortality, is too low to draw any conclusions.

### Medical care by a geriatric assessment team

Medical care by a geriatric assessment team compared to standard care with regard to use of drugs was tested in only one study including a single nursing home with 69 residents (Cavalieri 1993 [11]) (details in Table 5).

**Table 5:** Description of included primary studies for the comparison geriatric assessment team versus usual care

Study	Participants	Intervention	Comparison	Outcomes
**Cavalieri** **1993 **[11]	33 residents assigned to the intervention group and 36 residents to the control group in the same nursing home. Mean age: 82 years.	Aimed at identifying potential healthcare outcomes, e.g. reduction of prescription of drugs. A Comprehensive Geriatric Assessment Team: Team of geriatricians and geriatric nurse practitioners. The team evaluated each residents on arrival to the nursing home and was responsible for all medical treatment during the study period. **Extent of implementation**: Not reported.	Usual care: residents managed by individual physicians without formal training in geriatrics.	Number of drugs prescribed, hospital admissions/use of health care services.

### Drug use

Statistically significantly fewer drugs were prescribed for the intervention group at three months than for the control group (mean difference -2.3 drugs 95% CI -4.58 to -0.02).

### Health-related outcomes

There were no statistically significant differences between intervention and control groups in number of hospitalisations (mean 0.6 admissions in both groups) or number of days alive (274 days since study recruitment in the intervention group versus 235 days in the control group).

### Summary and quality of evidence

Medical care by a geriatric assessment team led to statistically and clinically significant fewer drugs being prescribed when compared to usual care. No statistically significant effect was demonstrated for the single health-related outcome measured, which was number of hospitalisations. However, the quality of the evidence was graded as very low and no conclusions about the effect of geriatric assessment teams may be drawn (GRADE summary of findings in Additional file 1, Table S8).

### Early psychiatric intervention by a psychogeriatric team

We identified only one study, conducted in 22 nursing homes with 106 residents, that examined the effect of early psychiatric intervention for use of psychotropic drugs and physical restraint (Kotynia-English 2005 [18]) (details in Table 6).

**Table 6:** Description of included primary studies for the comparison early psychiatric intervention versus usual care

Study	Participants	Intervention	Comparison	Outcomes
**Kotynia English 2005 **[18]	53 residents in the intervention group and 53 residents in the control group in 22 nursing homes in Australia. Mean age: 84 years.	Aimed at improving mental health and physical outcomes by early detection of symptoms, thereby indirectly reducing the need for psychotropic medication. Psychiatric assessment of all residents in the intervention group. Residents attaining a GDS-15 (Geriatric Depression Scale) score greater than 5 or a NPI (Neuropsychiatric Inventory) score greater than zero in any of its 12 sections were referred to a psychogeriatric multi disciplinary team for treatment and systematic follow up untill the problem was solved (usually ca 3 months). **Extent of implementation: **All the residents were screened but no other information than that residents were referred when needed.	Usual care, i.e. when residents screened positively, they were not automatically referred to psychiatric treatment but only if it was judged necessary.	Use of psychotropic agents or use of PRN medication, physical restraint.

### Drug use

There were no statistically significant differences between intervention and control groups in the use of psychotropics at 12 months (regular use RR 1.08 (95% CI 0.80 to 1.46) or in occasional use of psychotropics (RR 0.89 (95% CI 0.77 to 1.03)).

### Health-related outcomes

No statistically significant effect was found for use of physical restraint (RR 1.44 (95% CI 0.55 to 3.76)).

### Summary and quality of evidence

Early psychiatric intervention by a psycho-geriatric team did not demonstrate any statistically significant effect on use of drugs or physical restraint. The quality of the evidence was graded as very low and no conclusions about the effect may be drawn (GRADE summary of findings in Additional file 1, Table S9).

### Activity program for residents

One study conducted in a single nursing home with 81 residents examined the effect of a program for dementia care (Rovner 1996 [25]) (details in Table 7). The intervention consisted of an activity program for residents, guidelines for prescription of psychotropic drugs with transfer of prescribing responsibility from the general practitioner to the psychiatrist as well as one-hour meetings each week for six months between the psychiatrist and the activities staff to discuss each patient's behavioural, functional and medical status.

**Table 7:** Description of included primary studies for the comparison activity program versus usual care

Study	Participants	Interventions	Comparison	Outcomes
**Rovner** **1996 **[25]	42 residents in the intervention group and 39 residents in the control group in a 250 beds nursing home in USA. Mean age: 82 years.	Aimed at reducing behaviour disorders in residents with dementia. Program with three components: - Daily 5 hours activity program with music, exercise, crafts, relaxation, reminiscences, word games and food preparation - Guidelines for psychotropic drug management. Psychotropic drugs were considered as potentially inappropriate and the aim was to reduce their use. Prescribing were turned over to the psychiatrist. - Weekly 1 hour educational meetings between the study psychiatrist and activities staff that focused on patients' predisposing features to behavioural disorders. Each patient's behavioral, funtional and medical status were discussed. **Extent of implementation: **Thirty-eight of 42 (92.8%) intervention patients attended the activity program daily. Three patients and one patient's family refused participation. On average, intervention patients spent 17.0 (5.9) hours per week in the activity program. Activity levels for controls were observed four times during the 6 month period (months 2, 3, 5, 6) and revealed that an average of 23.4% (5.1) participated in nursing home provided activities. At 6 months, intervention patients were more than 10 times more likely to participate in activities than were controls (OR = 13.71; 95% CI [4.50, 41.73]; P <.001).	Usual care, i.e. each resident had about 3 to 6 activity hours each week: discussion group, arts and crafts, special programs with outside entertainers and bedside sensory stimulation. The physician could make contact with the psychiatrist if needed. Usual care was modified by the intervention: when intervention residents participated in acitivites elsewhere, the nurse to resident ratio increased in the nursing unit.	Number of residents using antipsychotics, number of drugs, use of physical restraint.

### Drug use

At six months follow-up there were no statistically significant differences between the intervention and control groups for the use of antipsychotic drugs (RR 0.52 (95% CI 0.26 to 1.04)) or number of drugs per resident (mean difference 0.60 (95% CI -0.90 to 2.10)).

### Health-related outcomes

Physical restraints were significantly less used in the intervention group during activity, which were arranged outside the nursing unit, compared with controls (RR 0.49 (95% CI 0.25 to 0.95)). However, there was no statistically significant difference between the groups in physical restraint use when both groups stayed in the nursing unit (RR 0.65 (95% CI 0.39 to 1.09)).

### Summary and quality of evidence

An activity program for residents did not demonstrate any statistically significant effect on use of drugs, but did indicate a statistically significant effect in use of physical restraints during activity. However, the quality of the evidence was graded as very low and no conclusions about the effect of activity programs may be drawn (GRADE summary of findings in Additional file 1, Table S10).

## Discussion

In this review we identified, assessed and summarised evidence of the effect of interventions with a primary or secondary aim of reducing potentially inappropriate use of drugs in nursing homes. We identified 20 randomized controlled trials conducted in 431 nursing homes involving 14 416 residents and an unknown number of health personnel, but no systematic reviews that met our inclusion criteria. Three of our comparison categories (including a total of ten studies) compared an educational initiative of some kind with practice as usual, while one comparison category (including seven studies) tested medical review against usual practice. For the other three comparison categories geriatric teams, early psychiatric intervention and activity program for residents, we identifed only one study for inclusion in each category.

Five of the ten studies that tested the effect of educational initiatives reported a statistically significant effect for at least one outcome measure on the use of drugs. However, it was not possible to determine whether any of the three types of educational interventions (educational outreach initiatives, educational meetings alone or as a part of a complex intervention) had a better effect than the others. However, these studies represent only a small subset of the many studies of the effect of educational interventions in other environments involving health personnel. Systematic reviews with a broader scope than ours have concluded that there is a small, general effect on health professionals' practice and an even smaller effect on patient outcomes of all the three types of educational interventions examined in our review [32,33]. The quality of the evidence for these results was graded as moderate [33]. The effects, however, varied from study to study without obvious reasons. This may at least partly be explained by a complex interaction of the degree of implementation of the education programme, the intensity of the programme (which is usually very low in these kind of studies), teaching methods and their quality, characteristics of the organisation, how senior leaders and practitioners perceive the seriousness of the outcomes and the complexity of the desired change [33]. Educational interventions alone can probably not be expected to change behaviour when leaders and practitioners do not perceive it to be important, or when the change is complex and dependent on the interaction of many people [32,33]. There is reason to believe that these considerations also apply to educational programmes in nursing homes. In conclusion, the results indicate that educational programmes for health personnel may have a small effect on drug managing practice when circumstances are favourable. Although the quality of the evidence for these results in nursing homes varies from low to very low, the results are consistent with results from systematic reviews of a much more comprehensive evidence base [32,33].

Seven studies examined the effect of medication review on drug use. The role of pharmacists varied from doing a limited medication review with a passive response to the doctor, to teaching and coordination and involvement of other health care professionals in a multidisciplinary team. In four of the studies there was a statistically significant effect on at least one measure of drug use (Crotty 2004b [13], Crotty 2004c [14], Zermansky 2006 [28], Patterson 2010 [29]), whereas the results in the remaining three were non-significant. In two of these latter studies, the pharmacist did review the drugs, but the end product seemed to be a written recommendation to the doctor as the only follow-up. This was also the case for the pharmacist medication review performed in one of the studies that we classified as educational meetings with at least one additional intervention (Roberts 2001 [24]). In the third study without a statistically significant effect the pharmacist was active in a multidisciplinary team and presumably gave recommendations or feedback orally during the meetings, but without any medication review done beforehand (Schmidt 1998 [26]/Claesson 1998 [12]).

All interventions that were examined in this review are interventions known to be context dependent [9], meaning that they may work in some contexts and not in others. Contextual factors such as the success of implementation of the intervention programme, characteristics of the host organisation, local culture, degree of support by organisational leaders, expertise of the investigational staff as well as the staff targeted by the intervention, intensity and duration of the intervention and a wide range of other factors will influence the effectiveness of the intervention. It is, therefore, a general problem that interventions and settings are poorly described with regard to detail as well as to the extent of implementation. For example, only five out of twenty included studies gave some information related to the extent of implementation of the intervention (Crotty 2004b [14], Rovner 1996 [25], Stein 2001 [27], Kuske 2009 [19], Testad 2010 [30]). Consequently, reasons for variations in effects are difficult to explain. However, there are some findings from other reviews that could tentatively illuminate some of the sources of heterogeneity. For example, passive feedbacks in the form of written recommendations to the doctor can be interpreted both as a reduction of the medication review to an intervention consisting of printed material and as a rather feeble feedback. The effect of printed material as an intervention to change behaviour has been described as uncertain [34]. Similarly, a previous systematic review of audit and feedback interventions concluded that the effect was probably greater the more intensive the feedback [35]. Therefore, in either case, in these three studies the feedback and follow-up may have been too weak.

In conclusion, the results indicate that medication review with the participation of a clinical pharmacist may have a positive influence on the use of drugs in nursing homes. This conclusion is consistent with the more comprehensive evidence base in a systematic review of interventions to improve the medication of elderly in general [36]. However, even the quality of this larger evidence base for these results was graded as low.

For our other three comparison categories, geriatric assessment team (Cavalieri 1993 [11]), early psychiatric intervention (Kotynia-English 2005 [18]) and activity measures for the residents (Rovner 1996 [25]), there were only one study with few participants and of high or uncertain risk of bias in each comparison. The quality of the existing evidence is therefore too low to determine whether these interventions can influence drug use or not, and suggests a need for larger studies of better quality.

Some of the studies in this review only assessed the level of drug use without considering the appropriateness of the drugs prescribed and used. However, severe adverse effects have been shown to be associated with use of drugs like antipsychotics [37-39] and benzodiazepines [36]. Monitoring and reducing or changing the use of such drugs could therefore be an important goal in itself.

Our preselected primary outcomes were prescription and use of drugs. However, these outcomes are actually related to process, whereas the ultimate goals are improvement in quality of life for the residents and reduction of adverse events. Because of the demonstrated associations between intake of certain drugs and health outcomes it is generally assumed that by reducing these drugs health outcomes will be improved and adverse effects reduced. Accordingly, health outcomes would not be expected to improve if there is no effect on drug use. To the contrary, however, three studies indicated small but apparently real changes in health-related outcomes without any statistically significant change in medication (Furniss 2000 [17], Kuske 2009 [19], Rovner 1996 [25]). Perhaps other factors in the active intervention such as greater awareness among staff regarding their own behaviour could explain this. Of the nine studies that did detect a statistically significant effect on drug use, five measured at least one of our preselected health-related outcomes. Only one of these had a statistically significant effect on one of the health-related outcomes, i.e. number of falls per resident, although not for the total number of patients who fell (Zermansky 2006 [28]).

It is important to be aware of the fact that the boundaries between the categories we classified the studies by are not clear cut. For example, both Roberts 2001 [24] and Crotty 2004a [13] could have been classified as medication reviews rather than as composite educational intervention and educational outreach initiatives, respectively. Also, several of the interventions could have been classified as multifaceted but we wanted to emphasise what we thought was the main content. Likewise, interventions such as pharmacists working in a multidisciplinary team could have been classified as multidisciplinary.

### Possible weaknesses

Although our literature search conforms with the criteria for a systematic review and included screening for references in included studies, weaknesses intrinsic to any search strategy means that relevant studies may have been missed. We did not look for grey literature nor did we do any formal assessment of risk of publication bias. Also, in the screening process we may have overlooked studies if drug use was not reported in the abstract as one of the outcomes. In particular, this may apply to studies of different activity and environmental initiatives.

## Conclusions

Our review indicates that interventions using educational outreach, on-site education given alone or as part of an intervention package and pharmacist medication review under certain circumstances may reduce inappropriate drug use in nursing homes. The evidence for these results ranges from very low to low quality but is supported by findings from other reviews of studies from a wide range of health care settings. The quality of the evidence for the results from the three other interventions that were examined, medical care by a geriatric assessment team, early psychiatric intervention and activity programme for residents was graded as very low and no conclusions about their effect on drug use can be drawn. For the same reason, no conclusions may be drawn for the effect on health-related outcomes.

## Implications for research and practice

Further research should concentrate on improving our understanding of when interventions such as education or medication reviews are likely to be effective and how to improve them. Also, studies that examine the effect of combined approaches, conducted by a multidisciplinary team and structurally and organisationally integrated in the institution are likely to be of interest. Many of the studies described in this review were small or provided too little detail on design or how the interventions were carried out, with very low quality of evidence as a result. Therefore, it is important that further studies are sufficiently powered and take greater care in describing study and intervention conditions. Only then may conclusions be drawn on a reliable basis.

It has been pointed out that doctors, nurses and pharmacists receive little training in drug treatment of older people in their education [40]. Consequently, it is difficult to imagine any intervention for reducing drug use that does not imply some kind of educational initiative. Most likely, however, several measures of organisational and structural character would have to be planned as part of the managerial approach. This could comprise organisation of continuous education of relevant health personnel, allocation of time to make it possible for personnel to participate and explicit procedures and routines for medication review actively involving key personnel in a multidisciplinary team setting. Carrying out these activities should be endorsed and followed up by those professionally responsible in the nursing home.

## Competing interests

The authors declare that they have no competing interests.

## Authors' contributions

LF was involved in assessing abstracts and all full-text copies, assessing risk of bias of pre-selected outcomes in all included papers and drafted and revised the manuscript. MCE was involved in assessing abstracts and all full-text copies, assessing risk of bias of pre-selected outcomes in all included papers and in revision of the final manuscript. EG was involved in screening of abstracts and in revision of the final manuscript. GEV was involved in grading of evidence and in revision of the final manuscript and acted as the third person for consultancy in disagreements between two reviewers. All authors read and approved the final manuscript.

## Pre-publication history

The pre-publication history for this paper can be accessed here:

http://www.biomedcentral.com/1471-2318/11/16/prepub

## Supplementary Material

Additional file 1 **Supplementary Figures and Tables**. Figure S1: Flow chart of study selection. Table S1: Search strategy. Table S2: Excluded studies table. Table S3: Risk of bias assessments. Table S4: Grade summary of findings table - educational outreach. Table S5: Grade summary of findings table - educational meetings. Table S6: Grade summary of findings table - educational meetings with other co-interventions. Table S7: Grade summary of findings table - medication review. Table S8: Grade summary of findings table - geriatric assessment team. Table S9: Grade summary of findings table - early psychiatric intervention. Table S10: Grade summary of findings table - activity program for nursing home residents with dementia Click here for file
