# Supplementary material for: Effect of interventions to reduce potentially inappropriate use of drugs in nursing homes: a systematic review of randomised controlled trials
Source: BMC Geriatr. 2011 Apr 17;11:16. doi: 10.1186/1471-2318-11-16 (PMC3108292; doi:10.1186/1471-2318-11-16)
Supplement: Additional file 1 — Supplementary Figures and Tables. Figure S1: Flow chart of study selection. Table S1: Search strategy. Table S2: Excluded studies table. Table S3: Risk of bias assessments. Table S4: Grade summary of findings table - educational outreach. Table S5: Grade summary of findings table - educational meetings. Table S6: Grade summary of findings table - educational meetings with other co-interventions. Table S7: Grade summary of findings table - medication review. Table S8: Grade summary of findings table - geriatric assessment team. Table S9: Grade summary of findings table - early psychiatric intervention. Table S10: Grade summary of findings table - activity program for nursing home residents with dementia [file 1471-2318-11-16-S1.PDF]

Contents

Table S1: Search strategy 2

Table S2: Excluded studies table 6

Table S3: Risk of bias assessments 9

Table S4: Grade summary of findings table - Educational outreach 23

Table S5: Grade summary of findings table - Educational meetings 24

Table S6: Grade summary of findings table - Educational meetings with other co-interventions 26

Table S7: Grade summary of findings table - Medication review 28

Table S8: Grade summary of findings table - Geriatric assessment team 31

Table S9: Grade summary of findings table - Early psychiatric intervention 32

Table S10: Grade summary of findings table - Activity program for nursing home residents with dementia 33

Table S1: Search strategy

Database: Cochrane Library
Date: 25.8.09
Number of hits: 450

ID	Search
#1	MeSH descriptor **Nursing Homes** explode all trees
#2	MeSH descriptor **Residential Facilities**, this term only
#3	MeSH descriptor **Homes for the Aged**, this term only
#4	MeSH descriptor **Assisted Living Facilities**, this term only
#5	MeSH descriptor **Long-Term Care**, this term only
#6	nursing next home*:ti,ab
#7	long-term next care:ti,ab
#8	long-term next facilit*:ti,ab
#9	(#2 OR #4 OR #5 OR #7 OR #8)
#10	MeSH descriptor **Aged** explode all trees
#11	(elderly or old or older or aged or geriatr*):ti,ab
#12	(#10 OR #11)
#13	(#9 AND #12)
#14	(#1 OR #3 OR #6)
#15	(#13 OR #14)
#16	MeSH descriptor **Drug Utilization** explode all trees
#17	MeSH descriptor **Drug Toxicity** explode all trees
#18	MeSH descriptor **Drug Therapy** explode all trees
#19	MeSH descriptor **Drug Interactions** explode all trees
#20	MeSH descriptor **Psychotropic Drugs** explode all trees
#21	MeSH descriptor **Central Nervous System Agents** explode all trees
#22	MeSH descriptor **Therapeutic Uses** explode all trees
#23	(inappropriat* near prescri*):ti,ab
#24	(suboptimal near prescri*):ti,ab
#25	(multiple near medication):ti,ab
#26	(unnecessary near drug*):ti,ab
#27	drug next therapy:ti,ab
#28	(reduc* near drug*):ti,ab
#29	(drug* near use):ti,ab
#30	(drug* near prescri*):ti,ab
#31	polypharmacy:ti,ab
#32	(appropriat* near medication*):ti,ab
#33	(prescribing near quality):ti,ab
#34	(pharmaceutical or pharmacological):ti,ab

#35	(sedative* or tranquillizers or sedation):ti,ab
#36	MeSH descriptor **Pharmaceutical Preparations** explode all trees
#37	MeSH descriptor **Drug Monitoring** explode all trees
#38	(drug* near monitor*):ti,ab
#39	(drug* near screen*):ti,ab
#40	MeSH descriptor **Drug Administration Schedule** explode all trees
#41	MeSH descriptor **Central Nervous System Depressants** explode all trees
#42	(drug* near withdrawal*):ti,ab
#43	(#16 OR #17 OR #18 OR #19 OR #20 OR #21 OR #22 OR #23 OR #24 OR #25 OR #26 OR #27 OR #28 OR #29 OR #30 OR #31 OR #32 OR #33 OR #34 OR #35 OR #36 OR #37 OR #38 OR #39 OR #40 OR #41 OR #42)
#44	(#43 AND #15)

Database: Ovid MEDLINE(R) 1950 to August Week 4 2009
Date: 3.9.09
Number of hits: 394
1. exp nursing homes/ or intermediate care facilities/ or skilled nursing facilities/
2. nursing home$.tw.
3. homes for the aged/
4. or/1-3
5. residential facilities/
6. Long-Term Care/
7. intermediate care.tw.
8. (extended care adj4 facilit$).tw.
9. skilled nursing facilit$.tw.
10. (long term care or long term facilit$).tw.
11. or/5-10
12. exp aged/ or exp "aged, 80 and over"/ or exp frail elderly/
13. (elderly or old or older or aged or geriatr$).tw.
14. 12 or 13
15. 11 and 14
16. 4 or 15
17. drug utilization/ or "drug utilization review"/
18. Drug Toxicity/
19. drug therapy/ or drug prescriptions/ or medication errors/
20. Drug Interactions/
21. exp Central Nervous System Agents/
22. exp therapeutic uses/
23. ((inappropriate or suboptimal) adj4 prescri$).tw.
24. (multiple adj4 medication).tw.
25. (unnecessary adj4 drug$).tw.
26. drug therapy.tw.
27. (reduc$ adj6 (drug or drugs)).tw.
28. (drug$ adj3 "use").tw.
29. (drug$ adj6 prescri$).tw.
30. (drug$ adj4 monitor$).tw.
31. (drug$ adj4 screen$).tw.
32. polypharmacy/
33. polypharmacy.tw.
34. (medication adj4 appropriateness).tw.
35. (prescribing adj4 quality).tw.
36. (pharmaceutical$ or pharmacological).tw.
37. (drug$ adj4 withdrawal).tw.
38. (sedative$ or tranquillizers or sedation).tw.
39. drug therapy.fs.

40. exp Pharmaceutical Preparations/
41. exp Pharmaceutical Preparations/
42. or/17-41
43. 16 and 42
44. limit 43 to "reviews (specificity)"
45. randomized controlled trial.pt.
46. controlled clinical trial.pt.
47. randomized.ab.
48. placebo.ab.
49. randomly.ab.
50. trial.ab.
51. groups.ab.
52. 45 or 46 or 47 or 48 or 49 or 50 or 51
53. humans.sh.
54. 52 and 53
55. 54 and 43
56. (2005$ or 2006$ or 2007$ or 2008$ or 2009$).ed,ep,yr,dp.
57. 55 and 56
58. 44 or 57

Database: EMBASE 1980 to 2009 Week 35
Date: 3.9.09
Number of hits: 205
1. nursing home/
2. home for the aged/
3. nursing home$.tw.
4. residential home/
5. long term facilit$.tw.
6. intermediate care facilit$.tw.
7. extended care facilit$.tw.
8. skilled nursing facilit$.tw.
9. or/4-8
10. exp aged/
11. (elderly or old or aged or older or geriatr$).tw.
12. geriatrics/ or gerontopsychiatry/
13. or/10-12
14. 9 and 13
15. nursing home patient/
16. (or/1-3) or 15
17. 14 or 16
18. exp "drug use"/
19. drug utilization/ or "utilization review"/
20. exp pharmacology/
21. exp drug therapy/
22. exp hypnotic agent/ or exp neuroleptic agent/ or exp antidepressant agent/ or exp central nervous system agents/ or exp anticonvulsive agent/ or exp drug screening/ or exp analgesic agent/ or exp sedative agent/
23. exp drug toxicity/
24. exp drug interaction/
25. exp analgesic agent/
26. ((inappropriate or suboptimal) adj4 prescri$).tw.
27. (multiple adj4 medication).tw.
28. (unnecessary adj4 drug$).tw.
29. drug therapy.tw.
30. (reduc$ adj6 (drug or drugs)).tw.
31. (drug$ adj4 monitor$).tw.
32. (drug$ adj4 screen$).tw.
33. (drug$ adj3 "use").tw.
34. (drug$ adj6 prescri$).tw.
35. (drug$ adj4 withdrawal).tw.

36. polypharmacy.tw.
37. (medication adj4 appropriateness).tw.
38. (prescribing adj4 quality).tw.
39. (pharmaceutical$ or pharmacological).tw.
40. exp pharmaceutics/
41. (sedative$ or tranquillizers or sedation).tw.
42. (medication adj4 inappropriat$).tw.
43. (medication adj4 "use").tw.
44. or/18-43
45. 17 and 44
46. limit 45 to "reviews (1 term high specificity)"
47. limit 45 to "treatment (1 term high sensitivity)"
48. limit 47 to em=2005$
49. limit 47 to em=2006$
50. limit 47 to em=2007$
51. limit 47 to em=2008$
52. limit 47 to em=2009$
53. or/48-52
54. 53 or 46

Database: ISI Web of Knowledge, Timespan=All Years. Databases=SCI-EXPANDED, SSCI, A&HCI
Date: 3.9.09
Number of hits: 414

TS=(nursing home*) AND (TS=(polypharmacy) OR TS=(polypharmacotherapy) OR TS=(inappropriate))

CRD

#	1	MeSH Nursing Homes QUALIFIERS CL ST SN OG MA TD UT EXPLODE 1
#	2	nursing NEAR home*
#	3	#1 or #2

Table S2: Excluded studies table

Study	Reason for exclusion
Primary studies	
**Ballard C,** Powell I, James I, Reichelt K, Myint P, Potkins D, Bannister C, Lana M, Howard R, O'Brien J, Swann A, Robinson D, Shrimanker J, Barber R: **Can psychiatric liaison reduce neuroleptic use and reduce health service utilization for dementia patients residing in care facilities.** *Int J Geriatr Psychiatry* 2002, 17:140-145.	Not a randomised controlled trial
**Burns** A, Allen H, Tomenson B, Duignan D, Byrne J: **Bright light therapy for agitation in dementia: a randomized controlled trial.** *International psychogeriatrics / IPA* 2009, 21:711-721.	Does not measure drug use
**Garland** K, Beer E, Eppingstall B, O'Connor DW: **A comparison of two treatments of agitated behavior in nursing home residents with dementia: Simulated family presence and preferred music.** *Am J Geriatr Psychiatry* 2007, 15:514-521.	Does not measure drug use
**Gurwitz** JH, Field TS, Rochon P, Judge J, Harrold LR, Bell CM, Lee M, White K, LaPrino J, Erramuspe-Mainard J, Deflorio M, Gavendo L, Baril JL, Reed G, Bates DW: **Effect of computerized provider order entry with clinical decision support on adverse drug events in the long-term care setting.** *J Am Geriatr Soc* 2008, 56:2225-2233.	Does not measure drug use
**Hanlon** JT, Weinberger M, Samsa GP, Schmader KE, Uttech KM, Lewis IK, Cowper PA, Landsman PB, Cohen HJ, Feussner JR: **A randomized, controlled trial of a clinical pharmacist intervention to improve inappropriate prescribing in elderly outpatients with polypharmacy.** *Am J Med* 1996, 100:428-437.	Population: Excludes nursing homes residents
**Holliday-Welsh** DM, Gessert CE, Renier CM: **Massage in the Management of Agitation in Nursing Home Residents with Cognitive Impairment.** *Geriatr Nur (Lond)* 2009, 30:108-117.	Not a randomized controlled trial
**Hutt** E, Ruscin JM, Corbett K, Radcliff TA, Kramer AM, Williams EM, Liebrecht D, Klenke W, Hartmann S: **A multifaceted intervention to implement guidelines improved treatment of nursing home-acquired pneumonia in a state veterans home.** *J Am Geriatr Soc* 2006, 54:1694-1700.	Not a randomised controlled trial
**Judge** J, Field TS, Deflorio M, Laprino J, Auger J, Rochon P, Bates DW, Gurwitz JH: **Prescribers' responses to alerts during medication ordering in the long term care setting.** *J Am Med Inform Assoc* 2006, 13:385-390. DOI 10.1197/jamia.M1945.	Outcome: Does not measure drug use but physicians responses to alerts
**Khunti** K, Kinsella B: **Effect of systematic review of medication by general practitioner on drug consumption among nursing-home residents.** *Age Ageing* 2000, 29:451-453.	Design: Lack of control group
**Lorenzo** P, Bell C, Masaki K, Iwasaki W, Blanchette P: **Polypharmacy Improvement Adherence Project: Nursing Home Medication Cost Outcomes.** *J Am Geriatr* Soc 2009, 57:S147-S148.	Conference abstract

**Monette** J, Miller MA, Monette M, Laurier C, Boivin JF, Sourial N, Le Cruguel JP, Vandal A, Cotton-Montpetit M: **Effect of an educational intervention on optimizing antibiotic prescribing in long-term care facilities**. *J Am Geriatr Soc* 2007, 55:1231-1235.	Outcome: Measures adherence to guidelines for use of antibiotics
**Naughton** BJ, Mylotte JM, Ramadan F, Karuza J, Priore RL: **Antibiotic use, hospital admissions, and mortality before and after implementing guidelines for nursing home-acquired pneumonia.** *J Am Geriatr Soc* 2001, 49:1020-1024.	Outcome: Measures adherence to guidelines for use of antibiotics
**Olsson** IN, Curman B, Engfeldt P: **Patient focused drug surveillance of elderly patients in nursing homes.** Pharmacoepidemiol Drug Saf 2010, 19:150-157.	Not a randomized controlled trial
**Schwartz** DN, Abiad H, DeMarais PL, Armeanu E, Trick WE, Wang Y, Weinstein RA: **An educational intervention to improve antimicrobial use in a hospital-based long-term care facility.** *J Am Geriatr Soc* 2007, 55:1236-1242.	Not a randomised controlled trial
**Ulfvarson** J, Adami J, Ullman B, Wredling R, Reilly M, von Bahr C: **Randomized controlled intervention in cardiovascular drug treatment in nursing homes.** *Pharmacoepidemiol Drug Saf* 2003, 12:589-593.	Cardiovascular drugs, no relevant data

Reviews

**Castelino** RL, Bajorek BV, Chen TF: **Targeting suboptimal prescribing in the elderly: a review of the impact of pharmacy services.** *Ann Pharmacother* 2009, 43:1096-1106.	Population: Elderly in general; no subgroup analyses of nursing home residents
**Forbes** D, Culum I, Lischka AR, Morgan DG, Peacock S, Forbes J, Forbes S: **Light therapy for managing cognitive, sleep, functional, behavioural, or psychiatric disturbances in dementia.** *Cochrane Database of Systematic Reviews* 2009, Issue 4. Art. No.: CD003946. DOI: 10.1002/14651858.CD003946.pub3.	Does not measure use of drugs
**Furniss** L, Lloyd SK, Burns A: **Medication use in nursing homes for elderly people.** *Int J Geriat Psyciatry* 1998, 13:433-439.	Not a systematic review
**Gallagher** P, Barry P, O'Mahony D: **Inappropriate prescribing in the elderly.** *J Clin Pharm Ther* 2007, 32:113-121.	Not a systematic review
**Gurwitz** JH, Field TS, Rochon P, Judge J, Harrold LR, Bell CM, Lee M, White K, LaPrino J, Erramuspe-Mainard J, DeFlorio M, Gavendo L, Baril JL, Reed G, Bates DW: **Effect of computerized provider order entry with clinical decision support on adverse drug events in the long-term care setting.** *J Am Geriatr Soc* 2008, 56:2225-2233.	Does not measure use of drugs (adverse events)
**Herman** AD, Johnson TM, Ritchie CS, Parmelee PA: **Pain management interventions in the nursing home: a structured review of the literature.** *J Am Geriatr Soc* 2009, 57:1258-1267.	Not a systematic review

**Holland** R, Desborough J, Goodyer L, Hall S, Wright D, Loke YK: **Does pharmacist-led medication review help to reduce hospital admissions and deaths in older people? A systematic review and meta-analysis.** *Br J Clin Pharmacol* 2008, 65:303–316.	Population: Elderly in general; no subgroup analyses of nursing home residents
**Kaur** S, Mitchell G, Vitetta L, Roberts MS: **Interventions that can reduce inappropriate prescribing in the elderly: a systematic review.** *Drugs Aging* 2009, 26:1013-1028.	Population: Elderly in general; no subgroup analyses of nursing home residents
**Kuske** B, Hanns S, Luck T, Angermeyer MC, Behrens J, Riedel-Heller SG: **Nursing home staff training in dementia care: a systematic review of evaluated programs.** Int Psychogeriatr 2007, 19:818-841.	Review of in-service interventions, but does not measure reduction of drugs
**Lai** CKY, Yeung JHM, Mok V, Chi I: **Special care units for dementia individuals with behavioural problems.** *Cochrane Database of Systematic Reviews* 2009, Issue 4. Art. No.: CD006470. DOI: 0.1002/14651858. CD006470.pub2.	The three studies that measured psychotropic drug use were not randomized controlled trials
**Liu** GG, Christensen DB: **The continuing challenge of inappropriate prescribing in the elderly: an update of the evidence.** *J Am Pharm Assoc* 2002, 42:847-857.	Not a systematic review, elderly in general, other designs
**Nishtala** PS, McLachlan AJ, Bell JS, Chen TF: **Psychotropic prescribing in long-term care facilities: impact of medication reviews and educational interventions.** *Am J Geriatr Psychiatry* 2008, 16:621-632.	Not a systematic review: no critical appraisal of studies
**Opie** J, Rosewarne R, O'Connor DW: **The efficacy of psychosocial approaches to behaviour disorders in dementia: a systematic literature review.** *Aust N Z J Psychiatry* 1999, 33:789-799.	Does not measure use of drugs
**Rollason** V, Vogt N: **Reduction of polypharmacy in the elderly: a systematic review of the pharmacist.** Drugs Aging 2003, 20:817-832.	Population: Elderly in general; no subgroup analyses of nursing home residents
**Scott** I, Jayathissa S: **Quality of drug prescribing in older patients: is there a problem and can we improve it?** *Intern Med J* 2010, 40:7-18.	Not a systematic review
**Spinewine** A, Schmader KE, Barber N, Hughes C, Lapana KL, Swine C, Hanlon JT: **Appropriate prescribing in elderly people: how well can it be measured and optimised?** *Lancet* 2007, 370:173-184.	Population: Elderly in general; no subgroup analyses of nursing home residents
**Statens beredning för medicinsk utvärdering** (SBU). Äldres läkemedelsanvändning - hur kan den förbättras? : en systematisk litteraturöversikt. Stockholm: SBU, 2009.	Population: Elderly in general; no subgroup analyses of nursing home residents
**Verrue** CLR, Petrovic M, Mehuys E, Remon JP, Vander Stichele R: **Pharmacists' interventions for optimization of medication use in nursing homes : a systematic review.** Drugs Aging 2009, 26:37-49.	Not a systematic review: No critical appraisal of studies
**Zwarenstein** M, Goldman J, Reeves S: **Interprofessional collaboration: effects of practice-based interventions on professional practice and healthcare outcomes.** *Cochrane Database of Systematic Reviews* 2009, Issue 3. Art. No.: CD000072. DOI: 10.1002/14651858.CD000072.pub2.	Intervention specific (interprofessional collaboration) systematic review with only one study of relevance: Schmidt 1998 (which is included in our review)

Table S3: Risk of bias assessments

**Avorn 1992** [10]	A randomized trial of a program to reduce the use of psychoactive drugs in nursing homes	
**Entry**	**Judgement**	**Description**
Allocation concealment?	Unclear	Cluster trial. Not mentioned and judged as unclear because of limited information also about the randomisation algorithm.
Adequate sequence generation?	Unclear	"The 12 nursing homes identified for inclusion in the study were grouped into six pairs matched on the basis of size, type of ownership, and level of drug use. […] One institution in each pair was then randomly assigned to receive the experimental program."
Baseline balance?	Unclear	
Blinding? (Use of drugs)	Unclear	Not reported.
Blinding? (Clinical outcomes)	Yes	The residents' performance was tested by "a research assistant blinded to the study design and the nursing homes' group assignment." […] "All clinical assessments were obtained throughout the study by the same research assistant…"
Blinding of recruiters?	Unclear	Not reported.
Incomplete outcome data addressed for:		
Clusters?	Yes	
Health personnel?	Unclear	Not reported.
Residents?	Unclear	30 days follow-up after the 5-month intervention program: 81% of participants in the experimental group and 84% in the control group.
Free of selective reporting?	Unclear	No reason to suspect selective reporting.
Free of other bias:		
Unit of analysis error?	Yes	
If matched, adjusted for matching?	Yes	
**Unclear risk of bias in relevant outcomes**		

**Cavalieri 1993** [11]	Geriatric assessment teams in nursing homes: do they work?	
**Entry**	**Judgement**	**Description**
Adequate sequence generation?	Unclear	"…69 consecutive nursing home patients randomly assigned on arrival to team and nonteam conditions." ""…those who had no expressed choice of physician were randomly placed into one of the two groups…"
Allocation concealment?	Unclear	Not likely
Blinding?	Yes	"Charts on each of these pateints covering the 12-month

		period after admission were reviewed by a medical student.”
Incomplete outcome data addressed?	Unclear	3 months follow-up
Free of selective reporting?	Unclear	No reason to suspect selective reporting
Free of other bias?	Unclear	Potenital contamination due to all patients being residents in the same nursing home?

High risk of bias in relevant outcome(s)

**Crotty 2004 a** [13]	An outreach intervention to implement evidence based practice in residential care: a randomized controlled trial	
**Entry**	**Judgement**	**Description**
Adequate sequence generation?	Yes	“All randomisation was conducted using a computer-generated random allocation program …”
Allocation concealment?	Yes	.”…by a person external to the project”
Baseline balance?	No	“At baseline, residents from the intervention group were more likely to have…”
Blinding?	Yes	“ Case notes were audited by nurses blinded to allocation for demographic information, diagnoses and stroke risk factors. Medication charts were reviewed for prescription…”
Blinding of recruiters?	Unclear	Not reported.
Incomplete outcome data addressed?		
Clusters?	Yes	
Health personnel?	Unclear	
Residents?	No	Follow-up at 7 months: “22.5% who participated at the first audit were not available at the second due to variuous reasons including death and moving facility. The attrition rate was as high as 37% in some facilities but there were no overall differences in attrititon rate between the intervention and control facilities..”
Free of selective reporting?	Unclear	No reason to suspect selective reporting.
Free of other bias:		
Unit of analysis error?	Yes	“To account properly for the cluster randomisation design used, the facility was recognised as the primary sampling unit in all analysis.”
If matched, adjusted for matching?	Unclear	Not reported.
Other		Only 59% of the patients were cared for by the physicians in the study.

High risk of bias in relevant outcomes

**Crotty 2004 b** [14]	Does the addition of a pharmacist transition coordinator improve evidence-based medication management and health outcomes in older adults moving from the hospital to a long-term care facility? Results of a randomized controlled trial	
**Entry**	**Judgement**	**Description**
Adequate sequence generation?	Yes	"The study biostatistician provided a computer-generated allocation sequence that used block randomization and was stratified by hospital."
Allocation concealment?	Yes	Most likely: "Randomization was coordinated by a centralized hospital pharmacy service."
Blinding?	Yes	"… independent pharmacists who were blinded to study-group allocation assessed patients' medication charts and case notes."
Incomplete outcome data addressed ?	Unclear	8 weeks follow-up was 79 % for the experimental group and 81 % for the control group. They did include data for those having died or otherwise had dropped out of the study for the outcome 'hospital usage'.
Free of selective reporting?	Unclear	
Free of other bias?	Unclear	It may seem as the control group was a little older, had some more comorbidity and used some more medication. Baseline differences were corrected for.

Unclear risk of bias for relevant outcomes

**Crotty 2004 c** [15]	An outreach geriatric medication advisory service in residential aged care: a randomised controlled trial of case conferencing	
**Entry**	**Judgement**	**Description**
Adequate sequence generation?	Yes	*Facilities*: "Computer-generated random numbers were used …"
	Unclear	*Residents*: Ten of the 20 residents nominated from the intervention facilities were randomly allocated …"
Allocation concealment?	Yes	*Facilities*: "…by a researcher independent of the investigators to assign five facilities to each of the intervention and control groups."
	Yes	*Residents:* "…by the pharmacy department using sequential sealed opaque envelopes …"
Baseline balance?	No	Report no statistical differences but some numbers are different, e.g. MAI score at baseline.
Blinding?	Yes	"All residents in the study had their medication charts reviewed pre- and post-intervention by an independent pharmacist using the Medication Appropriateness Index (MAI)."
Blinding of recruiters?	Unclear	Not reported.
Incomplete outcome data addressed for		
Clusters?	Yes	

Health personnel?	Unclear	Not reported.
Residents?	Unclear	Follow-up at 3 months: 71% of residents at follow-up, proportions equal for the three groups, reason for loss: death.
Free of selective reporting?	Unclear	No reason to suspect selective reporting.
Free of other bias:		
Unit of analysis error?	Yes	"Because of the cluster randomisation design used, the facility was recognised as the primary sampling unit in all analysis."
If matched, adjusted for matching?		No matching was reported.

Unclear risk of bias in relevant outcomes

**Fossey 2006** [16]	Effect of enhanced psychosocial care on antipsychotic use in nursing home residents with severe dementia: cluster randomised trial	
**Entry**	**Judgement**	**Description**
Adequate sequence generation?	Yes	"… the trial's statistician (blind to the identity of the homes) classified two homes in each region as having low neuroleptic use and two as having high use.The statistician then randomly assigned the homes to intervention or control, strativied by region and baseline neuroleptic use. The allocations were computer generated using stratified block randomisation (fixed block size of two).."
Allocation concealment?	Yes	Cluster trial. See first sentence above.
Baseline balance?	Yes	"Personal and clinical characteristics of the residents in both arms were similar at baseline".
Blinding?	Yes	Assessors were blinded: "Assessments at 12 months were carried out by a psychology research assistant who had not been employed during the intervention period."
Blinding of recruiters?	Yes	"… the family carers of residents were asked to give consent for involvement of their relatives. Research assistants carried out baseline assessments before randomisation." We interpret this to recruitment taking place before randomisation.
Incomplete outcome data addressed?		
Clusters	Yes	At follow-up at 12 months all clusters were analysed.
Health personnel	Unclear	Not reported
Residents	Yes	About same turn-over of patients in both groups: 181 residents in intervention nursing homes and 168 in control. During the study 69 patients left the intervention group while 64 new residents joined; 54 residents left the control group while 56 residents joined. At post-test there were 176 residents in the intervention group and 170 residents in the control group.
Free of selective reporting?	Unclear	Probably
Free of other bias?		Probably
Unit of analysis error?	Yes	
If matched, adjusted for		

matching?	Unclear	Matching used, but adjustment not reported. Weighting used to adjust for stratification factors.

Low risk of bias in relevant outcomes

**Furniss 2000** [17]	Effects of a pharmacist's medication review in nursing homes	
**Entry**	**Judgement**	**Description**
Adequate sequence generation?	Yes	"Using computer-generated pseudorandom numbers, one home in each pair was randomly allocated to …"
Allocation concealment?	Yes	Cluster trial. Allocation not mentioned, but the homes seem to have been all assigned at once: "Homes were randomised at the start of the observation phase."
Baseline balance?	Yes	"The 14 homes contained 424 residents, of whom 330 (78%) agreed to participate. On average, residents in the control homes were slightly younger, and there were proportionally fewer residents int these homes." (Baseline differences were adjusted for in analysis.)
Blinding?	Unclear	"The initial assessments were made by the study pharmacist and the psychiatrist. The second and third assessments were carried out by six registered Mental Nurses trained in the administration of the instruments, to ensure consistency and reliability."
Blinding of recruiters?	Unclear	Not reported.
Incomplete outcome data addressed?		
Clusters	Unclear	
Health personnel	Unclear	
Residents	Unclear	84 % follow-up at 8 months
Free of selective reporting?	Unclear	Probably
Free of other bias?		
Unit of analysis error?	Yes	"To account properly for the cluster randomisation design used, "nursing home' was forced into all regression models as a cluster variable (thus the unit of analysis was nursing home, not individual resident)."
If matched, adjusted for matching?	Unclear	Matched, but not adjusted for matching.

Unclear risk of bias in relevant outcomes

**Kotynia-English 2005** [18]	A randomized trial of early psychiatric intervention in residential care: impact on health outcomes	
**Entry**	**Judgement**	**Description**
Adequate sequence generation?	Yes	"The study was designed as a randomized, single-blinded, controlled trial. A random list of numbers was generated by computer and maintained centrally by an independent member of staff. Participants were allocated randomly to the intervention or usual care groups in random blocks of eight (four in each group)."

Allocation concealment?	Yes	See above.
Blinding?	Yes	"The researcher who carried out the assessment of all participants was blinded to group allocation."
Incomplete outcome data addressed?	No	Follow-up at 6 and 12 months. Follow-up of relevant outcomes: 69/106 (65%) – more residents died in the intervention group than in control.
Free of selective reporting?	Unclear	Probably.
Free of other bias?	Unclear	Some differences in baseline values. Patients from the same nursing home – contamination?

Unclear risk of bias in relevant outcomes

**Kuske 2009** [19]	Training in dementia care: a cluster-randomized controlled trial of a training program for nursing home staff in Germany	
**Entry**	**Judgement**	**Description**
Adequate sequence generation?	Yes	"… using a computer generated randomization list."
Allocation concealment?	Yes	Cluster trial: The allocation of the nursing homes to one of the three groups was conducted independently by an external statistical centre using a computer generated randomization list."
Baseline balance?	Unclear	
Blinding?	Yes	"Data analysts were not aware of the treatment conditons. Others: "Assessment instruments were administered either by a psychologist, a specialist in health and nursing or by trained and supervised research assistants. Sociodemographic characteristics of residents were gathered from the care documentation system while those of the staff were gathered by a short questionnaire." "…the outcomes concerning the residents were gathered from document sources with the system of care so that possible effects of the data collection (detection bias) were minimized."
Blinding of recruiters?	Unclear	Not reported.
Incomplete outcome data addressed?		
Clusters	Yes	
Health personnel	Unclear	60% follow-up.
Residents	Unclear	6 months follow-up: Comparable attrition in all three groups: 64%, 65% and 67%. Only those residents left seem to have been analysed.
Free of selective reporting?	Unclear	Probably
Free of other bias?		
Unit of analysis error?	Yes	
If matched, adjusted for matching?		No matching

Unclear risk of bias in relevant outcomes

**Loeb 2005** [20]	Effect of a multifaceted intervention on number of antimicrobial prescriptions for suspected urinary tract infections in residents of nursing homes: cluster randomised controlled trial.	
**Entry**	**Judgement**	**Description**
Adequate sequence generation?	Yes	"…we paired nursing homes within each province or state by size (number of occupied beds) and by proportion of residents with indwelling catheters. One member of each pair was randomised to the intervention and the other to usual care. A statistician independent of the study team used a random numbers table to assign the intervention to nursing homes (odd or even) corresponding to the number selected."
Allocation concealment?	Yes	Cluster trial: "Although allocation was concealed, given the nature of the intervention the nursing home staff could not be blinded to the intervention."
Baseline balance?	Yes	"The characteristics of the pairs of intervention and usual care nursing homes were similar at baseline".
Blinding?	Yes	"Pharmacies affiliated with the study (the source of confirmation of antimicrobial prescriptions) were, however, blinded."
Blinding of recruiters?	Unclear	Not reported.
Incomplete outcome data addressed?		
Clusters	Unclear	One cluster from each arm withdrew because of lack of nursing resuources (follow-up of 83% of clusters). Also, one cluster had missing data, resolved by collecting data for the first 6 months for this nursing home and the corresponding matched nursing home).
Health personnel	Unclear	
Residents	Unclear	Follow-up at 12 months: Follow-up of 88% of residents in intervention group and 90% in control.
Free of selective reporting?	Unclear	Probably
Free of other bias?		
Unit of analysis error?	Yes	"The unit of analysis was the nursing home".
If matched, adjusted for matching?	No	Adjustment for matching not reported.

Unclear risk of bias in relevant outcomes

**McCallion 1999** [21]	Educating nursing assistants to communicate more effectively with nursing home residents with dementia	
**Entry**	**Judgement**	**Description**
Adequate sequence generation?	Unclear	"One unit in each facility was randomly assigned to the NACSP condition and the other unit to a wait-list control (WC) condition." Partial cross-over trial.

Allocation concealment?	Unclear	Not reported.
Baseline balance?	Yes	“Significant differences were found, however, between treatment and control residents with respect to marital status and GDS scores.” “Significant differences between conditions were found at baseline on resident marital status and Global Deterioration Scale scores. To examine the impact of these variables three-way interaction effects were examined” (also done for characteristics of nursing homes).
Blinding?	Yes	“To the extent possible, data collectors were kept blind to the research hypotheses and to the intervention condition to which each NA and resident were assigned.”
Blinding of recruiters?	Unclear	Not reported.
Incomplete outcome data addressed for		
Clusters?	Yes	
Health personnel?	Unclear	Not reported
Residents?	Unclear	Follow-up at 3 and 6 months. Number not reported
Free of selective reporting?	Unclear	Probably
Free of other bias?		
Unit of analysis error?	No	Unit of analysis error in analysis in table 3
If matched, adjusted for matching?	Not applicable	

High risk of bias in relevant outcomes.

**Meador 1997** [22]	Predictors of antipsychotic withdrawal of dose reduction in a randomized controlled trial of provider education	
**Entry**	**Judgement**	**Description**
Adequate sequence generation?	Unclear	Cluster trial: “…facilities were paired by size and antipsychotic use and allocated randomly to the education and control groups….”
Allocation concealment?	Unclear	Not reported
Baseline imbalance?	Yes	“At baseline, residents of the educational and control homes had comparable demographic characteristics, levels of behavioral symptoms and patterns of psychotropic use.”
Blinding?	Yes	“The proportion of patients receiving antipsychotics […] was determined from medical records…”
Blinding of recruiters?	Unclear	Not reported.
Incomplete outcome data addressed?		
Clusters?	Yes	“All 12 randomized homes were included in this analysis.”
Health personnel?	Unclear	
Residents?	Unclear	6 months follow-up. 85% follow-up of residents in the intervention group and 91% of residents in control.
Free of selective reporting?	Unclear	Probably
Free of other bias?		Probably

Unit of analysis error?	Yes	“For the overall effect of the RCT, the unit of analysis was the nursing home…”.
If matched, adjusted for matching?	Unclear	Adjustment for matching not reported.

Unclear risk of bias in relevant outcome

**Midlöv 2002** [23]	Descriptive study and pharmacotherapeutic intervention in patients with epilepsy or Parkinson’s disease at nursing homes in southern Sweden	
**Entry**	**Judgement**	**Description**
Adequate sequence generation?	Unclear	Cluster trial: “… two thirds of the nursing homes were randomised (using Microsoft Excel 97) to active intervention, which meant that the advice was sent to the responsibel physician. The remaining nursing homes comprised the control group.
Allocation concealment?	Unclear	Not reported.
Baseline imbalance?	Unclear	
Blinding?	Yes	“To minimise bias, the pharmacists responsible for collection of data were blind to the outcome of the randomisation.”
Blinding of recruiters?	Unclear	Not reported.
Incomplete outcome data addressed?		
Clusters?	Unclear	Not reported.
Health personnel	Unclear	Not reported.
Residents	No	20% dropped out in the Parkinson intervention group, 15% in the control group. 12% dropped out in the epilepsy intervention group, 18% in the control group.
Free of selective reporting?	Unclear	No reason to supsect.
Free of other bias?		
Unit of analysis error?	Yes	“To account properly for the cluster randomisation design used, “nursing home”was forced into all regression models as a cluster variable..”
If matched, adjusted for matching?	Not applicable	No matching reported.

Unclear risk of bias in relevant outcomes

**Patterson 2010** [29]	An evaluation of an adapted U.S. model of pharmaceutical care to improve psychoactive prescribing for nursing home residents in Northern Ireland (Fleetwood Northern Ireland Study)	
**Entry**	**Judgement**	**Description**
Adequate sequence generation?	Yes	“The homes within the matched pairs were randomly assigned as intervention … or control … using a computer-generated table of random numbers.”
Allocation concealment?	Yes	“An independent researcher blinded to the identity of the homes undertook this.”

Baseline imbalance?	No	Imbalance in patient characteristics and "Baseline prescribing of these drugs was slightly higher in the intervention homes but not significantly so."
Blinding?	Yes	"One member of the research team (…) collected all data from intervention and control homes and GP practices as described above." The data for relevant outcomes were presumably collected from residents' nursing home records or from records kept as required for the regulatory body.
Blinding of recruiters?	Unclear	Not reported.
Incomplete outcome data addressed?		
Clusters	Unclear	1 home lost in control group, 1 home selected from 3 pairs of nursing homes previously randomized to serve as reserves. Does not state how this home was selected.
Health personnel	Unclear	
Residents	Yes	Follow-up immediately after end of intervention. 75% follow-up in experiment and 77% in control group. "AUC analysis, accounting for dropouts, produced similar results."
Free of selective reporting?	Unclear	No reason to suspect selective reporting.
Free of other bias?		
Unit of analysis error?	Yes	"P-values accounting for clustering", "mean differences accounting for clustering"
If matched, adjusted for matching?	Yes	

Unclear risk of bias in relevant outcomes

**Roberts 2001** [24]	Outcomes of a randomized controlled trial of a clinical pharmacy intervention in 52 nursing homes	
**Entry**	**Judgement**	**Description**
Adequate sequence generation?	Yes	"A randomization ratio of 1 (intervention):3 (control) was used …" […] Nursing homes were matched on resident age, RCI distribution, and bed number into groups of four homes after baseline data collection…" "Following matching, one home from each of the 13 clusters of four was drawn from a hat and independently assigned to the intervention group …"
Allocation concealment?	Yes	Cluster trial: "one home from each of the 13 clusters of four was drawn from a hat and **independently** assigned to the intervention group …"
Baseline balance?	Yes	
Blinding?	Yes	Data were collected from patient charts and a database for subsidized drugs (a 6% random sample showed an overall reproducibility of 97%) so we do not think this introduced bias.
Blinding of recruiters?	Unclear	Not reported.
Incomplete outcome data addressed?		
Clusters?	Yes	

Health personnel	Unclear	Not reported
Residents	Unclear	Follow-up at 12 months (after intervention started): 67% in intervention group and 69% in control.
Free of selective reporting?	Unclear	Probably
Free of other bias?		
Unit of analysis error?	Yes	"The nursing home was used as the unit of analysis for all prescription data."
If matched, adjusted for matching?	Unclear	Not reported.

Unclear risk of bias in relevant outcomes

**Rovner 1996** [25]	A randomized trial of dementia care in nursing homes	
**Entry**	**Judgement**	**Description**
Adequate sequence generation?	Yes	"The allocation procedure was a fixed, uniform randomization scheme by computer algorithm." However, there are some unclear points: "After the first replication, controls who continued to meet entry criteria were eligible for the second replication. Fourteen of 20 (70%) controls were subsequently randomized. Intervention patients from the first replication were excluded because they no longer represented usual nursing home patients."
Allocation concealment?	Unclear	Not likely: Not mentioned
Blinding?	Yes	"The proportion of patients receiving antipsychotics […] was determined from medical records…"
Incomplete outcome data addressed?	Yes	6 months follow-up of 91 % of participants: "Eight patients dropped out before the trial began… […] One intervention and one control patient died during the trial and 3-month data were used".
Free of selective reporting?	Unclear	Probably
Free of other bias?	Unclear	It is unclear what risk of bias lies in this statement: "After the first replication, controls who continued to meet entry criteria were eligible for the second replication. Fourteen of 20 (70%) controls were subsequently randomized. Intervention patients from the first replication were excluded because they no longer represented usual nursing home patients."<br>Risk of contamination – all patients in the same home – which may lead to an underestimation of effect sizes.

Unclear risk of bias in relevant outcomes

**Schmidt 1998** [26] / **Claesson 1998** [12]	The impact of regular multidisciplinary team interventions on psychotropic prescribing in Swedish nursing homes / Drug use in Swedish nursing homes	
**Entry**	**Judgement**	**Description**
Adequate sequence generation?	Unclear	"Researchers randomly assigned one home in each pair to receive the intervention"

Allocation concealment?	Unclear	Cluster trial. It is not clear whether the nursing homes were allocated at the same time. The nursing homes were selected by the pharmacist group leader, while the project leader allocated them (and most likely had no detailed acquaintance with the different nursing homes, so this may not represent any serious bias). (Claesson 1998)
Baseline balance?	Unclear	"There were no significant differences in the demographic, funtional, or psychiatric characteristics of residents in experimental and control homes at baseline." (Schmidt 1998). However, the data reported in Claesson 1998 indicate that the nursing homes in the experimental group were smaller than nursing homes in the control group. Schmidt 1998: "To adjust for variations in facility size, each individual resident was weighted in proportion to the size of the nursing home."
Blinding?	Unclear	Not stated, but most likely based on clinical records.
Blinding of recruiters?	Not applicable "Informed consent was not obtained as the nature of the study was not regarded as influencing the residents."	
Incomplete outcome data addressed?		
Clusters?	No	3 out of 18 nursing home clusters were excluded/not eligible in the experimental group (Claesson 1998, not reported in Schmidt).
Health personnel	Unclear	
Residents	Unclear	Follow-up at one month after the 12 months intervention.The residents followed up were not the same as at pretest: Claesson 1998: Of the 626 residents in the intervention homes at pretest, 49% died (304) and 240 became new residents. No information on turnover in the control group, but there were 1228 residents at pretest and 1243 at posttest (Schmidt 1998).
Free of selective reporting?	Unclear	No reason to suspect this.
Free of other bias?		
Unit of analysis error?	No	Not corrected for intra cluster effect.
If matched, adjusted for matching?	No	No correction for matching. The authors make use of weighting to compensate for baseline differences, but that is not the same.

High risk of bias for in relevant outcomes

**Stein 2001** [27]	Educational program for nursing home physicians and staff to reduce use on non-steroidal anti-inflammatory drugs among nursing home residents	
**Entry**	**Judgement**	**Description**
Adequate sequence generation?	Unclear	"We attempted to match homes by number of beds to

		balance the numbers of control for difficult-to-measure factors associated with nursing home size. Because homes were recruited and randomized over a 6-month period this matching was only approximate. After the baseline evaluation of residents in each pair of homes was completed, the homes were randomly assigned to control or intervention."
Allocation concealment?	Unclear	Not mentioned, cluster trial, but the homes do not seem to have been all assigned at once.
Baseline balance?	Yes	
Blinding?	Yes	"Data collection was performed by a trained nurse masked to home assignment." (abstracted from medical records – we do not believe that this will introduce bias.)
Blinding of recruiters?	Yes	"After the baseline evaluation of residents in each pair of homes was completed, the homes were randomly assigned to control or intervention", i.e. recrutiment took place before randomisation.
Incomplete outcome data addressed?		
Clusters	Yes	
Health personnel?	Unclear	
Residents?	Yes	64% (76/119) follow-up at 3 months in intervention group, 59% (71/120) in control.
Free of selective reporting?	Unclear	Probably
Free of other bias?		
Unit of analysis error?	Yes	"Because the nursing home was the unit of randomization, all analyses account for the correlation between outcome variables for patients of a single facility."
If matched, adjusted for matching?	Unclear	Approximately matched, but no adjustments reported.

Unclear risk of bias in relevant outcomes

**Testad 2010** [30]	The effect of staff training on agitation and use of restraint in nursing home residents with dementia: a single-blind, randomized controlled trial	
**Entry**	**Judgement**	**Description**
Adequate sequence generation?	Unclear	"… we randomly assigned subjects at home level. One small and one larger home were randomly allocated to either intervention or the control condition…"
Allocation concealment?	Unclear	Not reported. Rated as unclear because of lack of information on the generation of the randomization sequence.
Baseline balance?	No	"… there were statistically significant differences in the proportions of residents using antipsychotics and restraints as well as total CMAI score between the groups…".
Blinding?	Yes	"The administration of the outcome measures and drug recording were done by a trained research nurse who was uninformed as to the objective and design of the study and

		the treatment allocation."
Blinding of recruiters?	Unclear	Not reported.
Incomplete outcome data addressed?		
Clusters	Yes	No clusters lost
Health personnel?	Unclear	"During the study period, there was considerable turnover of staff and at study end there were 56 (53.8 %) remaining in the intervention and 53 (57.0 % in the control group."
Residents?	Unclear	Follow-up immediately after intervention (6 months duration). Follow-up 66% in experimental group and 71% in control group, "attrition mainly due to death".
Free of selective reporting?	Unclear	No reason to suspect selective reporting.
Free of other bias?		
Unit of analysis error?	Yes?	Apparently used cluster as unit of analysis.
Matching?	No	Does not appear to adjust for matching

High risk of bias in relevant outcomes

**Zermansky 2006** [28]	Clinical medication review by a pharmacist of elderly people living in care homes – a randomised controlled trial	
**Entry**	**Judgement**	**Description**
Adequate sequence generation?	Yes	"Patients were randomised in randomly sized blocks of two to eight patients using an algorithm written in Visual basic in Microsoft Access"
Allocation concealment?	Unclear	Not reported.
Blinding?	Yes	"A trained nurse (blind to randomisation) assessed cognitive and physical functioning…"
Incomplete outcome data addressed?	Unclear	6 months follow-up was 84 % for both groups
Free of selective reporting?	Unclear	Probably
Free of other bias?	Unclear	Risk of contamination

Unclear risk of bias in relevant outcomes

Table S4: Grade summary of findings table - Educational outreach

Educational outreach compared to usual practice

Populasjon: Residents in nursing homes
Setting: Nursing homes
Intervensjon: Educational outreach
Sammenligning: Usual practice

Outcomes	Illustrative comparative risks* (95% CI)		Relative effect (95% CI)	No of Participants (studies)	Quality of the evidence (GRADE)	Comments
	Assumed risk	Corresponding risk				
	**Usual practice**	**Educational outreach**				
**Drug use:** Measured by calculation of score by a self-developed index for use of psychoactive drugs, possibly range 1-7<br>**Follow-up:** Mean 30 days	The mean use of drugs changed for the control group from **1.74 til 1.60** scores	Mean adjusted change for drug use in intervention group was **0.37 lower**<br>(0.08 til 0.67 lower)		678<br>(1 study: Avorn 1992)	⊕⊕OO<br>**low**[2]	Adjusted change score: -0,37 (95 % KI -0,08 to -0,67)
**Drug use:** Percentage of residents that discontinued antipsychotics<br>**Follow-up:** Mean 30 days	Percentage of residents that discontinued antipsychotics in the control group was **14 percent**	Percentage of residents that discontinued antipsychotics in the intervention group was **18 higher**<br>(3 to 33 higher)		678<br>(1 study: Avorn 1992)	⊕⊕OO<br>**low**[1,2]	
**Drug use:** Measured by number of prescriptions of psychotropic drugs<br>**Follow-up:** Mean 7 months	**680 per 1000**	**605 per 1000**<br>(469 to 782)	**RR 0.89**<br>(0.69 to 1.15)	715<br>(1 study: Crotty 2004a)	⊕OOO<br>**very low**[3]	
**Drug use measured as psychotropic drugs used regularly**<br>**Follow-up:** Mean 7 months	**488 per 1000**	**454 per 1000**<br>(400 to 512)	**RR 0.93**<br>(0.82 to 1.05)	715<br>(1 study: Crotty 2004a)	⊕OOO<br>**very low**[3]	
**Falls during previous 3 months**<br>**Follow-up:** Mean 7 months	**219 per 1000**	**256 per 1000**<br>(188 to 346)	**RR 1.17**<br>(0.86 to 1.58)	715<br>(1 study: Crotty 2004a)	⊕OOO<br>**very low**[3]	
**Physical restraint use**	See comments					Not measured in any of the studies
**Admission to hospital**	See comments					Not measured in any of the studies
**Mortality**	See comments					Not measured in any of the studies

*The basis for the **assumed risk** is the median control group risk across studies. The **corresponding risk** (and its 95% confidence interval) is based on the risk in the comparison group and the **relative effect** of the intervention (and its 95% CI).

CI: Confidence interval; **RR:** Risk ratio;

[1] Unclear risk of bias
[2] Only one study and a wide confidence interval
[3] High risk of bias

Table S5: Grade summary of findings table - Educational meetings

Educational meetings compared to usual practice

Populasjon: Residents in nursing homes
Setting: Nursing homes
Intervention: Educational meetings
Comparison: Usual practice

Outcomes	Illustrative comparative risks* (95% CI)		Relative effect (95% CI)	No of Participants (studies)	Quality of the evidence (GRADE)	Comments
	Assumed risk	Corresponding risk				
	**Usual practice**	**Educational meetings**				
**Drug use: Use of neuroleptics** Follow-up: Mean 12 months	Mean use of drugs (neuroleptics) in the control group was **42.1 prosent**	Mean use of drugs (neuroleptics) in the intervention group was **19.1 lower** (0.5 to 37.7 lower)		338 (1 study: Fossey 2006)	⊕⊕OO **low**[1]	
**Drug use:** Use of other psychotropics Follow-up: Mean 12 months	Mean use of drugs (other psychotropics) in the control group was **57 prosent**	Mean use of drugs (other psychotropics) in the control group was **5.9 higher** (5.5 lower to 27.2 higher)		336 (1 study: Fossey 2006)	⊕⊕OO **low** [1]	
**Drug use:** Use of sedative drugs (neuroleptics, hypnotics, tranquilizers) Follow-up: Mean 6 months	See comments	See comments		217 (1 study: Kuske 2009)	⊕OOO **very low** [1,2]	No statistically significant differences between groups
**Drug use:** Number of days prior week residents received antipsychotic, antianxiety, antidepressant medications Follow-up: Mean 6 months	Mean drug use: Number of days prior week residents received antipsychotic, antianxiety or antidepressant medications in the control group were **1.57 days**	Mean drug use: Number of days prior week residents received antipsychotic, antianxiety or antidepressant medications in the intervention group were **0.27 lower** (1.02 lower to 0.48 higher)		105 (1 study: McCallion 1999)	⊕OOO **very low** [1,3]	No statistically significant differences between groups
**Drug use**: Number of days prior week NSAIDs were used Follow-up: Mean 3 months	Mean drug use: Number of days prior week NSAIDs were used in the control group were **6.2 days**	Mean drug use: Number of days prior week NSAIDs were used in the intervention group were **4.3 lower** (6.41 to 2.19 lower)		147 (1 study: Stein 2001)	⊕OOO **very low** [1,2]	
**Drug use**: Number of days prior week acetaminophen were used Follow-up: Mean 3 months	Mean drug use: Number of days prior week acetaminophen were used in the control group were **2.1 days**	Mean drug use: Number of days prior week acetaminophen were used in the intervention group were **3.0 higher** (1.53 to 4.47 higher)		147 (1 study: Stein 2001)	⊕OOO **very low** [1,2]	
**Drug use**: Proportion of residents taking antipsychotic drugs Follow-up: mean 6 months and at 12 months	The mean proportion of residents taking antipsychotic drugs in the control groups was **14.3 percent**	The mean proportion of residents taking antipsychotic drugs in the intervention groups was 15 **higher** (0 to 0 higher: no data)		90 (1 study: Testad 2010)	⊕OOO **very low**[3,1]	Baseline differences. No statistically significant differences between groups neither at 6 nor at 12 months
**Falls** Follow-up: Mean12 months	Mean number of falls in the control group was in percent **55 percent**	Mean number of falls in the intervention group was in percent **2.6 lower** (18.7 lower to 23.8 higher)		346 (1 study: Fossey 2006)	⊕⊕OO **low** [1]	

**Physical restraint I** measured as proportion of residents subjected to physical restraint Follow-up: Mean 6 months	See comments	See comments		217 (1 study: Kuske 2009)	⊕OOO **very low** [1,2]	Statistical significant increase in use of physical restraints in the control group but not in intervention group
**Physical restraint II** measured as number of days used prior week Follow-up: Mean 6 months	See comments	See comments		105 (1 study: McCallion 1999)	⊕OOO **very low** [1,3]	No statistically significant differences between groups
**Physical restraint III** measured as proportion of residents subjected to restraints Follow-up: mean 6 months and at 12 months	The mean proportion of residents subjected to physical restraints in the control groups was **46 percent**	The mean proportion of residents subjected to physical restraints in the intervention groups was **2 higher** (CI not reported)		90 (1 study: Testad 2010)	⊕OOO **very low** [3,1]	Baseline differences. No statistically significant differences between groups neither at 6 nor at 12 months
**Admission to hospital**						Not measured in any of the studies
**Mortality**						Not measured in any of the studies

*The basis for the **assumed risk is** the control group risk. The **corresponding risk** (and its 95% confidence interval) is based on the assumed risk in the comparison group and the **relative effect** of the intervention (and its 95% CI).

CI: Confidence interval

[1] Wide confidence interval and only one study
[2] Unclear risk of bias
[3] High risk of bias

Table S6: Grade summary of findings table - Educational meetings with other co-interventions

Educational meetings and workshops with other co-interventions versus usual care						
Population: Residents in nursing homes<br>Settings: Nursing homes<br>Intervention: Educational meetings and workshops with other co-interventions<br>Comparison: Usual care						
**Outcomes**	**Illustrative comparative risks* (95% CI)**		**Relative effect (95% CI)**	**No of participants (studies)**	**Quality of the evidence (GRADE)**	**Comments**
	Assumed risk	Corresponding risk				
	**Usual care**	**Educational meetings and workshops with other co-interventions**				
**Number of prescriptions for antibiotics for suspected urinary tract infection**<br>Follow-up: Mean 12 months	Mean drug use: Number of prescriptions for antibiotics for suspected urinary tract infection per 1000 resident days in the control group was **1.59**	Mean drug use: Number of prescriptions for antibiotics for suspected urinary tract infection per 1000 resident days in the intervention group was<br>**0.49 lower**<br>(0.93 to 0.06 lower)		3754<br>(1 study: Loeb 2005)	⊕⊕OO<br>low[1]	
**Total number of prescriptions of antibiotics per 1000 residents day**<br>Follow-up: Mean 12 months	Mean drug use: Total number of prescriptions for antibiotics per 1000 resident days in the control group was<br>**3.93**	Mean drug use: Total number of prescriptions for antibiotics per 1000 resident days in the intervention group was<br>**0.37 lower**<br>(1.17 lower to 0.44 higher)		3754<br>(1 study: Loeb 2005)	⊕⊕OO<br>low [1,2]	
**Use of drugs: Number of days per 100 of antipsychotic use**<br>Follow-up: Mean 6 months	Mean drug use: Number of days per 100 of antipsychotic use in the control group was<br>**26.0 dager per 100**	Mean drug use: Number of days per 100 of antipsychotic use in the intervention group was **6.3 lower**<br>(6.55 to 6.05 lower)		1152<br>(1 study: Meador 1997)	⊕⊕OO<br>low [1,2]	During the study period, there was a 23% attributable reduction in days of antipsychotic use in the intervention homes (p=0,014).
**Use of drugs: Number of residents receiving any psychotropic medication**<br>Follow-up: Mean 12 months	**618 per 1000**	**562 per 1000**<br>(513 to 618)	**RR 0.91**<br>(0.83 to 1)	1328<br>(1 study: Roberts 2001)	⊕⊕OO<br>low [1,2]	
**Number of hospitalisations I (Loeb 2005)**<br>Follow-up: Mean 12 months	Rate of all cause admissions to hospital in the control group was<br>**0.81 per 1000 resident days**	Rate of all cause admissions to hospital in the control group was<br>**0.17 higher**<br>(0.14 lower to 0.48 higher)		3754<br>(1 study: Loeb 2005)	⊕⊕OO<br>low [1,2]	

**Number of hospitalisations II (Roberts 2001)**<br>Follow-up: Mean 12 months	See comments	See comments		2212<br>(1 study: Roberts 2001)	⊕⊕OO<br>low [1,2]	No statistical significant differences between groups:<br>Mean percentage change for the intervention group was<br>+1,30 (95 % CI -26,32 til 28,91)<br>For the control group: -16,86 (95 % KI -30,62 til 3,11)
**Mortality**<br>Follow-up: Mean 12 months	Mean number of deaths in the control group was **1.09 per 1000 resident days**	Mean number of deaths in the intervention group was **0.07 higher**<br>(0.22 lower to 0.36 higher)		3754<br>(1 study: Loeb 2005)	⊕⊕OO<br>low [1,2]	
**Mortality II (Roberts 2001)**<br>Follow-up: Mean 12 months			**HR 0.85**<br>(0.68 to 1.06)	2212<br>(1 study: Roberts 2001)	⊕⊕OO<br>low [1,2]	
**Falls**						Not measured in any of the studies
**Physical restraint**						Not measured in any of the studies

*The basis for the **assumed risk** is the control group risk. The **corresponding risk** (and its 95% confidence interval) is based on the assumed risk in the comparison group and the **relative effect** of the intervention (and its 95% CI).

CI: Confidence interval; **RR:** Risk ratio; **HR**: Hazard ratio

[1] Unclear risk of bias
[2] Wide confidence interval and only one study

Table S7: Grade summary of findings table - Medication review

Medication review compared to usual care

Population: Residents in nursing homes
Settings: Nursing homes
Intervention: Medication review
Comparison: Usual care

Outcomes	Illustrative comparative risks* (95% CI)		Relative effect (95% CI)	No of participants (studies)	Quality of the evidence (GRADE)	Comments
	Assumed risk	Corresponding risk				
	**Usual care**	**Medical review**				
**MAI-score (**Medication Appropriateness Index) **to assess quality of prescribing** Follow-up: Mean 8 weeks	MAI-score for quality of prescribing in the control group was **6.5 scores**	MAI-score for quality of prescribing in the intervention group was **4 lower** (6.76 to 1.24 lower)		88 (1 study: Crotty 2004b)	⊕OOO **very low**[1,2]	
**MAI-score** Follow-up: Mean 3 months	MAI-score for quality of prescribing in the control group was improved by a score of **0.4**	MAI-score for quality of prescribing in the intervention group was improved by **a score of 4,1 (95 % CI 2.1 to 6.1)**		100 (1 study: Crotty 2004c)	⊕OOO **very low**[1,2]	The MAI-score in the intervention group improved by 4.1 score (95 % CI 2.1 to 6.1) compared to control that had a mean change of 0.4 score (95 % CI -0.4 to 1.2) (p<0,001).
**Number of drugs** Follow-up: Mean 3 months	Mean number of drugs in the control group was **6.1**	Mean number of drugs in the intervention group was **0.60 lower** (1.96 lower to 0.76 higher)		100 (1 study: Crotty 2004c)	⊕OOO **very low**[1,2]	
**Number of drug prescriptions:** Follow-up: Mean 8 months	Number of drug prescriptions in the control group was **4.4**	Number of drug prescriptions in the intervention group was **0.5 lower** (0.04 lower to 1 higher)		276 (1 study: Furniss 2000))	⊕⊕OO **low**[1,2]	
**Number of drugs for residents with Parkinson** Follow-up: Mean 6 months	Mean number of drugs for residents with Parkinson in the control group was **8.2**	Mean number of drugs for residents with Parkinson in the intervention group was **0.2 higher** (2.39 lower to 2.79 higher)		69 (1 study: Midlöv 2002)	⊕OOO **very low**[1,2]	
**Number of drugs for residents with epilepsy** Follow-up: Mean 6 months	Mean number of drugs for residents with epilepsy in the control group was **8.7**	Mean number of drugs for residents with epilepsy in the intervention group was **0.5 lower** (1.36 lower til 0.36 higher)		63 (1 study: Midlöv 2002)	⊕OOO **very low**[1,2]	
**Number of prescriptions for psychotropics** Follow-up: Mean 1 month	**791 per 1000**	**767 per 1000** (728 to 815)	**RR 0.97** (0.92 to 1.03)	1805 (1 study: Schmidt 1998)	⊕OOO **very low**[2,3]	

**Number of prescriptions for antipsychotics** Follow-up: Mean 1 month	See comments	See comments		1805 (1 study: Schmidt 1998)	⊕OOO **very low**[2,3]	The outcome is reported as percentage change from before to after: 19% reduction of prescribing in the intervention group (p=0,007) and 7 % reduction in the control group (p=0,176).
**Number of prescriptions for non-recommended hypnotics** Follow-up: Mean 1 month	See comments	See comments		1805 (1 study: Schmidt 1998)	⊕OOO **very low**[2,3]	The outcome is reported as percentage change from before to after: 37% reduction of prescribing in the intervention group (p=0,001) and 3% reduction in the control group (n.s).
**Number of prescriptions for non-recommended anxiolytics** Follow-up: Mean 1 month	See comments	See comments		1805 (1 study: Schmidt 1998)	⊕OOO **very low**[2,3]	The outcome is reported as percentage change from before to after: 0% change of prescribing in the intervention group and 7% increase in the control group.
**Number of prescriptions for nonrecommended antidepressants** Follow-up: Mean 1 month	See comments	See comments		1805 (1 study: Schmidt 1998)	⊕OOO **very low**[2,3]	The outcome is reported as percentage change from before to after: 59% reduction of prescribing in the intervention group (p=0,001) og 34 % reduction in the control group (p=0,002).
**Number of drug changes** Follow-up: Mean 6 months	See comments	See comments		555 (1 study: Zermansky 2006)	⊕⊕OO **low**[1,2]	Relative ratio of means is reported: 1.34 (95 % CI 1.21 til 1.48).
**Number of drugs per patient** Follow-up: Mean 6 months	See comments	See comments		555 (1 study: Zermansky 2006)	⊕⊕OO **low**[1,2]	Relative ratio of means: 0.98 (95 % CI 0.92 til 1.04).
**Proportion of residents taking inappropriate psychoactive medications** Follow-up: mean 12 months	**500 per 1000**	**206 per 1000** (123 to 329)	**OR 0.26** (0.14 to 0.49)	252 (1 study: Patterson 2010)	⊕⊕OO **low**[1,2]	
**Use of health services (emergency visits and re-hospitalisations)** Follow-up: Mean 8 weeks	See comments	See comments	**RR 0.58** (0.28 to 1.21)	88 (1 study: Crotty 2004b)	⊕OOO **very low**[1,2]	Intention to treat analysis as a secondary analysis.
**Hospitalisations: Number of patients hospitalised during 6 months** Follow-up: Mean 6 months	**187 per 1000**	**170 per 1000** (114 to 245)	**OR 0.89** (0.56 til 1.41)	555 (1 study: Zermansky 2006)	⊕OOO **very low**[1,2]	
**Falls I (Crotty 2004b)** Follow-up: Mean 8 weeks	**364 per 1000**	**433 per 1000** (258 to 724)	**RR 1.19** (0.71 til 1.99)	88 (1 study: Crotty 2004b)	⊕OOO **very low**[1,2]	
**Falls II (Furniss 2000)**	See comments	See comments	Not estimable	276 (1 study: Furniss 2000)	⊕OOO **very low**[1,2]	Data not reported. Authors reported that there was no statistically significant difference between the two groups.

**Falls III: Number of patients falling during 6 months (Zermansky 2006)** Follow-up: Mean 6 months	**321 per 1000**	**257 per 1000** (191 til 334)	**OR 0.73** (0.50 til 1.06)	555 (1 study: Zermansky 2006)	⊕OOO **very low**[1,2]	
**Falls IV: Number of falls per patient during 6 months (Zermansky 2006)** Follow-up: Mean 6 months	See comments	See comments		555 (1 study: Zermansky 2006)	⊕OOO **very low**[1,2]	Ratio of means: 0.59 (95 % KI 0.49 til 0.70).
**Falls V (Patterson 2010): Fall rate** Follow-up: mean 12 months	The fall rate in the control groups was **11.4**	The fall rate in the intervention groups was **4.9 higher** (CI not reported)		252 (1 study: Patterson 2010)	⊕OOO **very low**[1,2]	During the study year the fall rate (number of falls per 100 person-months) was 11.4 in the control group compared to 16.3 in the intervention group (p=0.09).
**Mortality I (Furniss 2000)** Follow-up: Mean 8 months	See comments	See comments		276 (1 study: Furniss 2000)	⊕OOO **very low**[1,2]	4 deaths in the intervention group versus 14 deaths in the control group (p=0,028) in the intervention period, but not statistically significant differences for the total study period: 26 versus 28 deaths.
**Mortality II (Zermansky 2006)** Follow-up: Mean 6 months	**173 per 1000**	**157 per 1000** (105 til 228)	**OR 0.89** (0.56 til 1.41)	555 (1 study: Zermansky 2006)	⊕OOO **very low**[1,2]	

*The basis for the **assumed risk** is the control group risk. The **corresponding risk** (and its 95% confidence interval) is based on the assumed risk in the comparison group and the **relative effect** of the intervention (and its 95% CI).

C**I:** Confidence interval; **RR:** Risk ratio; OR: Odds ratio

[1] Unclear risk of bias
[2] Wide confidence interval and only one study
[3] High risk of bias

Table S8: Grade summary of findings table - Geriatric assessment team

Geriatric assessment team compared to usual care

Population: Residents in nursing homes
Settings: Nursing homes
Intervention: Geriatric assessment team
Comparison: Usual care

Outcomes	Illustrative comparative risks* (95 % CI)		Relative effect (95% CI)	No of participants (studies)	Quality of the evidence (GRADE)	Comments
	Assumed risk	Corresponding risk				
	**Usual care**	Geriatric assessment team				
**Mean number of drug prescriptions per resident** Follow-up: Mean 3 months	Mean number of drug prescriptions in the control group was **5.9**	Mean number of drug prescriptions in the intervention group was **2.3 lower** (4.58 to 0.02 lower)		71 (1 study: Cavalieri 1993)	⊕OOO **very low**[1,2]	
**Hospitalisation** Follow-up: Mean 3 months	See comments	See comments		71 (1 study: Cavalieri 1993)	⊕OOO **very low**[1,2]	No statistical significant difference: Mean number of hospitalisations in both groups was 0,6

*The basis for the **assumed risk is** the control group risk. The **corresponding risk** (and its 95% confidence interval) is based on the assumed risk in the comparison group and the **relative effect** of the intervention (and its 95% CI).

CI: Confidence interval

[1] High risk of bias
[2] Wide confidence interval and only one study

Table S9: Grade summary of findings table - Early psychiatric intervention

Early psychiatric intervention compared to usual care

Populasjon: Residents in nursing homes
Setting: Nursing homes
Intervention: Early psychiatric intervention
Comparison: Usual care

Outcomes	Illustrative comparative risks* (95% CI)		Relative effect (95% CI)	No of participants (studies)	Quality of the evidence (GRADE)	Comments
	Assumed risk	Corresponding risk				
	**Usual care**	**Early psychiatric intervention**				
**Number of residents using psychotropics** Follow-up: Mean 12 months	**684 per 1000**	**739 per 1000** (547 to 999)	**RR 1.08** (0.8 to 1.46)	69 (1 study: Kotynia-English 2005)	⊕OOO **very low**[1,2]	
**Number of residents using psychotropics as needed** Follow-up: Mean 12 months	**974 per 1000**	**867 per 1000** (750 to 1000)	**RR 0.89** (0.77 to 1.03)	70 (1 study: Kotynia-English 2005)	⊕OOO **very low**[1,2]	
**Physical restraint use** Follow-up: Mean 3 months	**154 per 1000**	**222 per 1000** (85 to 579)	**RR 1.44** (0.55 to 3.76)	75 (1 study: Kotynia-English 2005)	⊕OOO **very low**[1,2]	
**Falls**						Not measured
**Hospitalisations**						Not measured
**Mortality**						Not measured

*The basis for the **assumed risk** is the control group risk. The **corresponding risk** (and its 95% confidence interval) is based on the assumed risk in the comparison group and the **relative effect** of the intervention (and its 95% CI).

CI: Confidence interval; **RR:** Risk ratio;

[1] Unclear risk of bias
[2] Wide confidence interval and only one study

Table S10: Grade summary of findings table - Activity program for nursing home residents with dementia

Activity program for nursing home residents with dementia compared to usual care

Population: Residents in nursing homes
Settings: Nursing homes
Intervention: Activity program
Comparison: Usual care

Outcomes	Illustrative comparative risks* (95 % CI)		Relative effect (95% CI)	Antall deltagere (studier)	Quality of the evidence (GRADE)	Comments
	Assumed risk	Corresponding risk				
	**Usual care**	**Activity program**				
**Number of residents using antipsychotics**<br>Follow-up: Mean 6 months	**410 per 1000**	**213 per 1000**<br>(107 to 426)	**RR 0.52**<br>(0.26 to 1.04)	81<br>(1 study: Rovner 1996)	⊕OOO<br>very low[1,2]	
**Total number of drugs used**<br>Follow-up: Mean 6 months		Total number of drugs used in the intervention group was **0.60 higher** than in the control group (0.90 lower to 2.10 higher)		81<br>(1 study: Rovner 1996)	⊕OOO<br>very low[1,2]	
**Physical restraintuse during activity**<br>Follow-up: Mean 6 months	**472 per 1000**	**231 per 1000**<br>(118 til 448)	**RR 0.49**<br>(0.25 to 0.95)	75<br>(1 study: Rovner 1996)	⊕OOO<br>very low[1,2]	
**Physical restraint use in the nursing unit**<br>Follow-up: Mean 6 months	**526 per 1000**	**342 per 1000**<br>(205 til 573)	**RR 0.65**<br>(0.39 to 1.09)	79<br>(1 study: Rovner 1996)	⊕OOO<br>very low[1,2]	
**Falls**						Not measured
**Hospitalisations**						Not measured
**Mortality**						Not measured

*The basis for the **assumed risk** is the control group risk. The **corresponding risk** (and its 95% confidence interval) is based on the assumed risk in the comparison group and the **relative effect** of the intervention (and its 95% CI).

CI: Confidence interval; **RR:** Risk ratio;

[1] Unclear risk of bias
[2] Wide confidence interval and only one study
